# Supplementary material for: Estimating a panel MSK dataset for comparative analyses of national absorptive capacity systems, economic growth, and development in low and middle income countries
Source: PLoS One. 2022 Oct 20;17(10):e0274402. doi: 10.1371/journal.pone.0274402 (PMC9584427; doi:10.1371/journal.pone.0274402)
Supplement: S1 Table — (DOCX) [file pone.0274402.s001.docx]

**Supporting Information**

**S1 Table: Handling Missing Data Strategies, Assumptions, Advantages and Disadvantages**

| **Strategies** | **Definition** | **Assumption** | **Advantage** | **Disadvantage** |
| --- | --- | --- | --- | --- |
| **Listwise deletion** | Complete-case analysis). It removes all data for a case that has any missing values [1,2] | MCAR | -Generally used if the researcher is performing a treatment study and wishes to compare a completers analysis (listwise deletion) vs. an intent-to-treat analysis (includes cases with missing data imputed or considered in a treatment design)  - Can be applied to any statistical model (structural equation modeling, multi-level regression, etc.)  - In the instance of MAR among independent variables (i.e., they do not depend on the values of dependent variables), listwise deletion parameter estimates can be unbiased [3] | - MCAR assumptions are generally rare to support  - Produce bias parameters and the estimates |
| **Pairwise deletion** | Available-case analysis aims to reduce the loss that occurs in listwise deletion. Pairwise maximizes all data available through checking into the correlation matrix between variables [1,4] | MCAR | -It increases statistical power in analyses  - Could be used in linear models such as linear regression, factor analysis, or SEM. | - Produce under- or overestimated standard of errors  - If the data mechanism is MAR, pairwise will return biased estimates. |
| **Mean substitution** | This method substitutes the mean value of a variable for missing value [1,5]. Also called unconditional mean substitution | NA | -Simple to execute | - Does not preserve the relationships among variables  - Leads to underestimated standard errors |
| **Regression imputation** | Called as conditional mean imputation, here missing value is based (regressed) on other variables [5] | -MCAR or MAR | -Maintain the relationship with other variables  - If the data are MCAR, least-squares coefficients estimates will be consistent and unbiased in large samples [6] | - No variability left  -Treated data as if they were collected  - Leads to underestimated standard errors & overestimated test statistics |
| **Cold deck imputation** | Cold Deck picks value from a case that has similar values on other variables [7] | MAR | -Easy to execute | -Removes the desired random variation |
| **Maximum likelihood (ML)** | It models the missing data based on observed data. This procedure considers available data as part of some distribution. Subsequently, parameters are estimated that maximize the chance of observing the observed data [8] | -MAR and Monotonic (meaning, that if an observation’s is missing on one variable, then the following variables of that observation have also missing data | - Consistent  -Asymptotically efficient (becomes efficient for large sample)  -Asymptotically normal | - ML can usually handle linear models, log-linear models. However, beyond that, ML still is lacking in theory and software implementation |
| **Expectation- Maximization Algorithm** | Similar to ML, but it is an iterative process. In the Expectation stage, data is imputed from observed data. In the second stage, the values are checked if they are the most likely. If not, it imputes again a more likely value [8] | MAR | - Easy to use  - Preserves the relationship with other variables | - Standard errors of the coefficients are incorrect (biased usually downward - underestimate)  -Models with overidentification, the estimates will not be efficient |
| **Multiple imputation**  **(many ways to execute MI)**  **-Multivariate Normal MI**  **-Chained MI - (Predictive Mean Matching, Regression, Logistic)** | MI replaces missing values with a set of imputed values. Analyses are subsequently performed on all the imputed values, and results are pooled [9] | MAR | -Consistent  -Asymptotically  efficient  -Asymptotically normal  - MI can be applied to any model, unlike ML, which can be applied only to limited models | - MI delivers a little different result in various runs. By seeding, the problem can be evaded.  - Some MI methods may cause unlikely values (e.g., negative values)  - Not all MI methods can handle heteroskedastic data |

**References**

1. Kang H. The prevention and handling of the missing data. Korean J Anesthesiol. 2013;64: 402–406. doi:10.4097/kjae.2013.64.5.402

2. Donner A. The Relative Effectiveness of Procedures Commonly Used in Multiple Regression Analysis for Dealing with Missing Values. Am Stat. 1982;36: 378–381. doi:10.1080/00031305.1982.10483055

3. Little RJA. Regression With Missing X’s: A Review. J Am Stat Assoc. 1992;87: 1227–1237. doi:10.2307/2290664

4. Kim J-O, Curry J. The Treatment of Missing Data in Multivariate Analysis. Sociol Methods Res. 1977;6: 215–240. doi:10.1177/004912417700600206

5. Zhang Z. Missing data imputation: focusing on single imputation. Ann Transl Med. 2016;4. doi:10.3978/j.issn.2305-5839.2015.12.38

6. Gourieroux C, Monfort A. On the Problem of Missing Data in Linear Models. Rev Econ Stud. 1981;48: 579–586. doi:10.2307/2297197

7. Haukoos JS, Newgard CD. Advanced Statistics: Missing Data in Clinical Research—Part 1: An Introduction and Conceptual Framework. Acad Emerg Med. 2007;14: 662–668. doi:https://doi.org/10.1111/j.1553-2712.2007.tb01855.x

8. Enders CK. TEACHER’S CORNER A Primer on Maximum Likelihood Algorithms Available for Use With Missing Data. 2001.

9. Little RJA, Rubin DB. The Analysis of Social Science Data with Missing Values. Sociol Methods Res. 1989;18: 292–326. doi:10.1177/0049124189018002004
