# Supplementary material for: Estimating a panel MSK dataset for comparative analyses of national absorptive capacity systems, economic growth, and development in low and middle income countries
Source: PLoS One. 2022 Oct 20;17(10):e0274402. doi: 10.1371/journal.pone.0274402 (PMC9584427; doi:10.1371/journal.pone.0274402)
Supplement: S2 Table — (DOCX) [file pone.0274402.s002.docx]

**Supporting Information**

**S2 Table. List of all 64 Variables, their Definitions, Sources, Missingness Amount in Observed Variables, and Acceptance/Rejection Status for the MSK Dataset**

|  |  | **Definition and source of the variables included in the MSK Database** |  |  |  |
| --- | --- | --- | --- | --- | --- |
|  |  |  |  |  |  |
| **Capacity** | **Variable code** | **Definition** | **Source** | **%Missing** | **Accept/Reject** |
| **Technology Capacity** | tippay | **Charges for the use of intellectual property, payments (BoP, current US$)**. Payment or charges per authorized use of intangible, non-produced, non-financial assets and proprietary rights (such as patents, trademarks, copyrights, industrial processes and designs including trade secrets, and franchises) and for the use, through licensing agreements, of produced originals of prototypes. Data are in current US dollars | IMF, World Bank | 33.50% | Accepted |
|  | tinddesapprebyco | **Industrial design applications, resident, by count** | WIPO | 75.12% | Rejected |
|  | tscitjar | **Scientific and technical journal articles.** Number of scientific and engineering articles published in the following fields: physics, biology, chemistry, mathematics, clinical medicine, biomedical research, engineering and technology, and earth and space sciences, per million people. | World Bank | 6.67% | Accepted |
|  | trandd | **Research and development expenditure (% of GDP)** | UNESCO | 81.71% | Accepted |
|  | tresinrandd | **Researchers in R&D (per million people)** | UNESCO | 87.97% | Accepted |
|  | ttechinrandd | **Technicians in R&D (per million people)** | UNESCO | 88.29% | Accepted |
|  | tpatappre | **Patent applications, residents** | WIPO | 60% | Rejected |
|  | ttradappresbyco | **Trademark applications, resident, by count** | WIPO | 70.89% | Rejected |
|  | thigexperofmanex | **High-technology exports (% of manufactured exports).** High-technology exports are products with high R&D intensity, such as in aerospace, computers, pharmaceuticals, scientific instruments, and electrical machinery. | UN, COMTRADE | 55.53% | Accepted |
|  | tsecedvoc | **Secondary education, vocational pupils.** Secondary students enrolled in technical and vocational education programs, including teacher training. | UNESCO | 53.58% | Accepted |
|  | teciscore | **ECI Score.** Measure of economic complexity containing information about both the diversity of a country's export and their sophistication. High ECI Score shows that an economy exports many goods that are of low ubiquity and that are produced by highly diversified countries. In other words, diverse and sophisticated economies have high scores. | OEC, MIT | 27.48% | Accepted |
|  |  |  |  |  |  |
| **Capacity** | **Variable code** | **Definition** | **Source** | **%Missing** | **Accept/Reject** |
| **Financial Capacity** | fdaystoenfctt | **Time required to enforce a contract (days).** Days required to enforce a contract, whereas the days are counted from the day a plaintiff files the lawsuit in court until payment. Low values indicate high competitiveness and vice verca. | World Bank, Doing Business Project | 6.18% | Accepted |
|  | fdomcrprsecbybkpergdp | **Domestic Credit by Banking Sector.** This includes all credit to various sectors (monetary authorities, banks, financial corporations) on a gross basis, with the exception of credit to the central government, which is net, as a % of GDP. | IMF, World Bank | 10.57% | Accepted |
|  | fopenind | **Openness Indicator.** (Import + Export)/GDP. Constant US 2010. | World Bank | 31.14% | Accepted |
|  | fdepcombkp1k | **Depositors with commercial banks (per 1,000 adults)** | IMF, World Bank | 47.32% | Rejected |
|  | fdaystoregpro | **Time required to register property (days).** The number of calendar days needed for businesses to secure rights to property. | World Bank, Doing Business Project | 10.24% | Accepted |
|  | fcosbstpropergni | **Cost of business start-up procedures (% of GNI per capita)** | World Bank | 6.18% | Accepted |
|  | ftaxrpergdp | **Tax revenue (% of GDP).** Tax revenue means compulsory transfers to the government for public purposes. | IMF, World Bank | 52.60% | Accepted |
|  | fcombkbr1k | **Commercial bank branches (per 100,000 adults)** | IMF, World Bank | 10.65% | Accepted |
|  | fdaystoobtelecconn | **Time to obtain electrical connection (Days).** Days to obtain electrical connection. Days experienced to obtain an electrical connection from the day an establishment applies for it to the day it receives the service. | World Bank, Enterprise Survey | 87.56% | Accepted |
|  | ftdaystobusi | **Time required to start a business (Days).** The number of days needed to complete the procedures to legally operate a business. | World Bank, Doing Business Project | 6.18% | Accepted |
|  | faccownperofpop15p | **Account ownership at a financial institution or with a mobile-money-service provider (% of pop ages 15+).** Account denotes the percentage of respondents who report having an account (by themselves or together with someone else) at a bank or another type of financial institution or report personally using a mobile money service in the past 12 months (% age 15+). | Demirguc-Kunt et al., 2018, Global Financial Inclusion Database, World Bank. | 86.99% | Accepted |
|  | fnewbusdenper1k | **New business density (new registrations per 1,000 people ages 15-64)**. New businesses registered are the number of new limited liability corporations registered in the calendar year. | World Bank, Enterprise Survey | 52.60% | Accepted |
|  |  |  |  |  |  |
| **Capacity** | **Variable code** | **Definition** | **Source** | **%Missing** | **Accept/Reject** |
| **Human Capacity** | hprimenrollpergross | **School enrollment, primary (% gross).** Ratio of total enrollment, regardless of age, to the population of the age group that officially corresponds to the primary level. | UNESCO | 25.93% | Accepted |
|  | hsecenrollpergross | **School enrollment, secondary (% gross).** Ratio of total enrollment, regardless of age, to the population of the age group that officially corresponds to the secondary level. | UNESCO | 42.20% | Accepted |
|  | hcompeduyears | **Compulsory education, duration (years).** No. of years that children are legally obliged to attend school. | UNESCO | 16.42% | Accepted |
|  | hgvtexpedupergdp | **Government expenditure on education (% of GDP).** General government expenditure on education (current, capital, and transfers) is expressed as a percentage of GDP. | UNESCO | 50% | Accepted |
|  | hpupteapriratio | **Primary pupil-teacher ratio**. Ratio (number of pupils enrolled in primary school) / (number of primary school teachers) | UNESCO | 38.94% | Accepted |
|  | hempinduspertotem | **Employment in industry (% of total employment).** Employment is defined as persons of working age who were engaged in any activity to produce goods or provide services for pay or profit, whether at work during the reference period or not at work due to temporary absence from a job, or to working-time arrangement. The industry sector consists of mining and quarrying, manufacturing, construction, and public utilities (electricity, gas, and water), in accordance with divisions 2-5 (ISIC 2) or categories C-F (ISIC 3) or categories B-F (ISIC 4). | ILO, World Bank | 8.54% | Accepted |
|  | hempserpertotem | **Employment in services (% of total employment).** Employment is defined as persons of working age who were engaged in any activity to produce goods or provide services for pay or profit, whether at work during the reference period or not at work due to temporary absence from a job, or to working-time arrangement. The services sector consists of wholesale and retail trade and restaurants and hotels; transport, storage, and communications; financing, insurance, real estate, and business services; and community, social, and personal services, in accordance with divisions 6-9 (ISIC 2) or categories G-Q (ISIC 3) or categories G-U (ISIC 4). | ILO, World Bank | 8.54% | Accepted |
|  | hprimcompra | **Primary completion rate, total (% of relevant age group)** | UNESCO | 40.24% | Accepted |
|  | hhciscale0to1 | **Human capital index (HCI) (scale 0-1).** The HCI calculates the contributions of health and education to worker productivity. The final index score ranges from zero to one and measures the productivity as a future worker of child born today relative to the benchmark of full health and complete education. | World Bank | 87.48% | Accepted |
|  | hlfwithadedu | **Labor force with advanced education (% of total working-age population with advanced education)** | ILO, World Bank | 78.46% | Accepted |
|  | hlfwithbasiced | **Labor force with basic education (% of total working-age population with basic education)** | ILO, World Bank | 78.13% | Rejected |
|  | hlfwithintermeded | **Labor force with intermediate education (% of total working-age population with basic education)** | ILO, World Bank | 78.13% | Rejected |
|  |  |  |  |  |  |
| **Capacity** | **Variable code** | **Definition** | **Source** | **%Missing** | **Accept/Reject** |
| **Infrastructural Capacity** | ielecconkwhpercapita | **Electric power consumption (kWh per capita).** Production of power plants and combined heat and power plants less transmission, distribution, and transformation losses and own use by heat and power plants. | IEA, World Bank | 66.42% | Rejected |
|  | icarrierdepwdwide | **Air transport, registered carrier departures worldwide**. Registered carrier departures worldwide are domestic takeoffs and takeoffs abroad of air carriers registered in the country | World Bank | 37.40% | Rejected |
|  | imobsubper100 | **Mobile cellular subscriptions (per 100 people).** | International Telecom Union, World Bank | 0.89% | Accepted |
|  | itelesubper100 | **Fixed telephone subscriptions (per 100 people)** | International Telecom Union, World Bank | 0.98% | Accepted |
|  | ibdbandsubper100 | **Fixed broadband subscriptions (per 100 people)** | International Telecom Union, World Bank | 9.43% | Accepted |
|  | iaccesselecperpop | **Access to electricity (% of population).** The percentage of population with access to electricity. | World Bank, Sustainable Energy for All | 7.72% | Accepted |
|  | ienergyusepercap | **Energy use (kg of oil equivalent per capita).** The use of primary energy before transformation to other end-use fuels, which is equal to indigenous production plus imports and stock changes, minus exports and fuels supplied to ships and aircraft engaged in international transport. | IEA, World Bank | 61.71% | Accepted |
|  | ieletanddislossesperoutput | **Electric power transmission and distribution losses (% of output)** | IEA, World Bank | 67.32% | Rejected |
|  | imachtpeqpervaladdmanu | **Machinery and transport equipment (% of value added in manufacturing).** Value added in manufacturing is the sum of gross output less the value of intermediate inputs used in production for industries classified in ISIC major division D. Machinery and transport equipment correspond to ISIC divisions 29, 30, 32, 34, and 35. | UNIDO, World Bank | 75.28% | Rejected |
|  | iindintperpop | **Individuals using the internet (% of population).** Internet users are individuals who have used the Internet (from any location) in the last 3 months. The Internet can be used via a computer, mobile phone, personal digital assistant, games machine, digital TV etc. | International Telecom Union, World Bank | 1.71% | Accepted |
|  | iraillinestotalkm | **Rail lines (total route km).** Railway route in km for train service, irrespective of the number of parallel tracks. | International Union of Railway | 78.05% | Rejected |
|  | isecinterserper1mill | **Secure internet servers per 1 million people** | World Bank | 35.93% | Rejected |
|  | iagmachtracper100sqkm | **Agricultural machinery, tractors per 100 sq. km of arable land** | FAO, World Bank | 98.05% | Rejected |
|  | ilpiquoftratraninfr | **Logistics performance index: Quality of trade and transport-related infrastructure (1=low to 5=high)**. Logistics professionals' perception of country's quality of trade and transport related infrastructure (e.g. ports, railroads, roads, information technology), on a rating ranging from 1 (very low) to 5 (very high). Scores are averaged across all respondents. | World Bank | 69.76% | Accepted |
|  |  |  |  |  |  |
| **Capacity** | **Variable code** | **Definition** | **Source** | **%Missing** | **Accept/Reject** |
| **Public Policy Capacity** | pcpiapsmgandinscl1to6 | **CPIA public sector management and institutions cluster average (1=low to 6=high).** The public sector management and institutions cluster includes property rights and rule-based governance, quality of budgetary and financial management, efficiency of revenue mobilization, quality of public administration, and transparency, accountability, and corruption in the public sector. | World Bank, CPIA Database | 7.97% | Accepted |
|  | pcpiastpolclavg1to6 | **CPIA structural policies cluster average (1=low to 6=high).** The structural policies cluster includes trade, financial sector, and business regulatory environment | World Bank, CPIA Database | 7.97% | Accepted |
|  | pstrengthoflegalright | **Strength of legal rights index (0=weak to 12=strong).** Strength of legal rights index measures the degree to which collateral and bankruptcy laws protect the rights of borrowers and lenders and thus facilitate lending. The index ranges from 0 to 12, with higher scores indicating that these laws are better designed to expand access to credit. | World Bank, Doing Buisness Project | 54.07% | Accepted |
|  | iscapscoravg | **Overall level of statistical capacity (scale 0 - 100).** A composite score (on a scale of 0-100) which assesses the capacity of a country’s statistical system in three areas (25 criteria): methodology; data sources; and periodicity and timeliness. | World Bank | 1.95% | Accepted |
|  | pcpiaeconmgtcl1to6 | **CPIA economic management cluster average (1=low to 6=high).** The economic management cluster includes macroeconomic management, fiscal policy, and debt policy. | World Bank, CPIA Database | 7.97% | Accepted |
|  |  |  |  |  |  |
| **Capacity** | **Variable code** | **Definition** | **Source** | **%Missing** | **Accept/Reject** |
|  | scpiabdhumanres1to6 | **CPIA building human resources rating (1=low to 6=high).** Building human resources assesses the national policies and public and private sector service delivery that affect the access to and quality of health and education services, including prevention and treatment of HIV/AIDS, tuberculosis, and malaria. | World Bank, CPIA Database | 7.97% | Accepted |
| **Social Capacity** | scpiaeqofpbresuse1to6 | **CPIA equity of public resource use rating (1=low to 6=high).** Equity of public resource use assesses the extent to which the pattern of public expenditures and revenue collection affects the poor and is consistent with national poverty reduction priorities | World Bank, CPIA Database | 7.97% | Accepted |
|  | scpiasocprorat1to6 | **CPIA social protection rating (1=low to 6=high).** Social protection and labor assess government policies in social protection and labor market regulations that reduce the risk of becoming poor, assist those who are poor to better manage further risks, and ensure a minimal level of welfare to all people. | World Bank, CPIA Database | 8.29% | Accepted |
|  | scpiapolsocinclcl1to6 | **CPIA policies for social inclusion/equity cluster average (1=low to 6=high).** The policies for social inclusion and equity cluster includes gender equality, equity of public resource use, building human resources, social protection and labor, and policies and institutions for environmental sustainability | World Bank, CPIA Database | 8.29% | Accepted |
|  | scovofsocprolbrpro | **Coverage of social protection and labor programs (% of population).** Coverage of social protection and labor programs (SPL) shows the percentage of population participating in social insurance, social safety net, and unemployment benefits and active labor market programs | World Bank | 87.48% | Rejected |
|  | sginiinedxwbest | **GINI index (World Bank estimate).** Measures income inequality. A Gini index of 0 represents perfect equality, while an index of 100 implies perfect inequality. | World Bank | 80.16% | Rejected |
|  | spovheadcnational | **Poverty headcount ratio at national poverty lines (% of population).** National poverty headcount ratio is the percentage of the population living below the national poverty line(s) | World Bank | 80.98% | Accepted |
|  | smultipovertyintensity | **The average share of weighted deprivations (intensity).** | World Bank | 97.97% | Rejected |
|  | ssocialconperofrev | **Social contributions (% of revenue).** Social contributions include social security contributions by employees, employers, and self-employed individuals, and other contributions whose source cannot be determined. They also include actual or imputed contributions to social insurance schemes operated by governments | IMF, World Bank | 53.74% | Accepted |
|  | smultipoverindex | **Multidimensional poverty index (scale 0-1).** Proportion of the population that is multidimensionally poor adjusted by the intensity of the deprivations | World Bank | 98.78% | Rejected |
|  |  |  |  |  |  |
