# Supplementary material for: Estimating a panel MSK dataset for comparative analyses of national absorptive capacity systems, economic growth, and development in low and middle income countries
Source: PLoS One. 2022 Oct 20;17(10):e0274402. doi: 10.1371/journal.pone.0274402 (PMC9584427; doi:10.1371/journal.pone.0274402)
Supplement: S3 Table — (DOCX) [file pone.0274402.s003.docx]

**Supporting Information**

**S6 Table. Comparative Ranking of Countries as Per Absorptive Capacity Index (2019)**

| **Rank** | **Country** | **Tech_Index** | **Finance_Index** | **Infrastructure_Index** | **HumanCapacity_Index** | **PublicPolicy_Index** | **SocialCapacity_Index** | **AbsorptiveCapacity_Index** |
| --- | --- | --- | --- | --- | --- | --- | --- | --- |
| 1 | Vietnam | 1.835127 | 1.823812 | 1.932174 | 0.765487 | 1.076316 | 0.504371 | 1.322881 |
| 2 | India | 4.306017 | 0.982093 | 0.825784 | 0.428893 | -0.11115 | 0.561976 | 1.165602 |
| 3 | Bosnia and Herzegovina | 0.230912 | 0.842245 | 2.37799 | 1.014441 | 0.332861 | 1.059924 | 0.976395 |
| 4 | Kosovo | 1.237119 | 0.593937 | 1.921194 | 0.621909 | 0.626732 | 0.436277 | 0.906195 |
| 5 | Moldova | 0.387072 | 0.462939 | 2.018654 | 0.425024 | 1.032233 | 1.019524 | 0.890908 |
| 6 | Georgia | 0.378321 | 0.507115 | 2.306922 | 0.393636 | 1.286522 | 0.438898 | 0.885235 |
| 7 | Mongolia | 1.928538 | 0.183368 | 0.108906 | 1.180915 | 0.670887 | 0.814918 | 0.814589 |
| 8 | Uzbekistan | 0.811956 | -0.2022 | 1.691056 | 0.950817 | 0.510404 | 0.976198 | 0.789705 |
| 9 | Bolivia | 0.293038 | 0.894145 | 1.058183 | 1.012945 | 0.155349 | 1.158961 | 0.762103 |
| 10 | St. Vincent and the Grenadines | 1.053247 | 0.208629 | 1.541172 | 0.660106 | 0.48422 | 0.26156 | 0.701489 |
| 11 | Grenada | 0.3357 | 0.14374 | 2.150201 | 0.598516 | 0.417394 | 0.514369 | 0.69332 |
| 12 | Armenia | 0.128657 | 0.107966 | 1.320656 | 0.568944 | 1.224323 | 0.48454 | 0.639181 |
| 13 | St. Lucia | 0.461946 | 0.093167 | 1.815016 | 0.638062 | 0.351249 | 0.457002 | 0.636074 |
| 14 | Dominica | 0.132727 | 1.010012 | 1.471019 | 0.758585 | 0.462641 | -0.14163 | 0.61556 |
| 15 | Kyrgyz Republic | 0.42882 | -0.21069 | 0.918591 | 0.663019 | 0.973312 | 0.717389 | 0.581741 |
| 16 | Cabo Verde | -0.23006 | 0.358551 | 0.900362 | 0.568667 | 0.370873 | 1.104725 | 0.512186 |
| 17 | Samoa | -0.37554 | 0.426603 | 0.845797 | 0.439965 | 1.087011 | 0.596361 | 0.503366 |
| 18 | Kenya | 0.820638 | 0.242582 | 0.088082 | 0.345713 | 0.817826 | 0.390348 | 0.450865 |
| 19 | Nepal | 0.123604 | 0.321283 | 0.715523 | 0.39143 | 0.584091 | 0.486709 | 0.437107 |
| 20 | Bhutan | -0.23118 | 0.578921 | 0.435955 | 0.466053 | 0.606513 | 0.527948 | 0.397369 |
| 21 | Honduras | 0.254915 | 0.323002 | 0.605793 | 0.342447 | 0.229982 | 0.594319 | 0.391743 |
| 22 | Cambodia | 0.074192 | 0.763915 | 0.704527 | 0.409797 | 0.388614 | -0.11648 | 0.370761 |
| 23 | Sri Lanka | -0.19002 | 0.233907 | 0.967374 | 0.555663 | 0.282689 | 0.148645 | 0.333043 |
| 24 | Rwanda | 0.327286 | -0.46087 | 0.051823 | -0.06512 | 1.26666 | 0.750217 | 0.311665 |
| 25 | Nigeria | 0.4944 | -0.31568 | 0.777391 | 0.117993 | -0.00503 | 0.630286 | 0.283226 |
| 26 | Maldives | -0.45821 | 0.245947 | 1.475443 | 0.492052 | -0.15242 | 0.00429 | 0.267849 |
| 27 | Lao PDR | 0.143695 | 0.577372 | 0.567417 | 0.261909 | -0.24236 | 0.269928 | 0.262993 |
| 28 | Senegal | -0.0125 | -0.25093 | 0.39761 | -0.24882 | 0.847148 | 0.458069 | 0.198429 |
| 29 | Tonga | -0.41569 | -0.09632 | 0.519016 | 0.309611 | 0.603783 | 0.101094 | 0.170249 |
| 30 | Ghana | -0.37397 | -0.55224 | 0.604351 | 0.353584 | 0.650955 | 0.182034 | 0.144118 |
| 31 | Tanzania | 0.026761 | -0.09467 | 0.203206 | -0.48162 | 0.207781 | 0.796058 | 0.109586 |
| 32 | Cote d'Ivoire | -0.07358 | -0.22658 | 0.366575 | -0.01271 | 0.480156 | 0.108809 | 0.107113 |
| 33 | Ethiopia | 0.577417 | -0.20974 | -0.22005 | -0.11129 | 0.017556 | 0.565574 | 0.103244 |
| 34 | Djibouti | 0.225756 | -0.15364 | 0.400645 | -0.04848 | 0.154949 | -0.04464 | 0.089098 |
| 35 | Lesotho | 0.303178 | 0.38123 | 0.19675 | -0.11242 | 0.142388 | -0.403 | 0.084688 |
| 36 | Togo | -0.03731 | -0.28685 | -0.00302 | 0.256051 | 0.283281 | 0.29095 | 0.083851 |
| 37 | Bangladesh | -0.10129 | 0.4261 | 0.06743 | 0.329799 | -0.23315 | -0.04416 | 0.074122 |
| 38 | Guyana | -0.3607 | -0.37911 | 1.019161 | 0.422247 | -0.21172 | -0.0783 | 0.068595 |
| 39 | Pakistan | 0.062532 | -0.09652 | 0.137691 | 0.111673 | 0.095186 | 0.099132 | 0.068282 |
| 40 | Kiribati | 0.019126 | 0.386583 | 0.227063 | 0.653179 | -0.60597 | -0.39131 | 0.048111 |
| 41 | Vanuatu | -0.19003 | -0.04284 | 0.385256 | 0.219316 | 0.447211 | -0.55403 | 0.044149 |
| 42 | Burkina Faso | 0.135966 | -0.13823 | -0.15245 | 0.123046 | 0.317112 | -0.04392 | 0.040255 |
| 43 | Benin | -0.16928 | -0.15866 | -0.3173 | -0.00672 | 0.570117 | 0.148464 | 0.011105 |
| 44 | Malawi | -0.1415 | -0.29775 | -0.33264 | 0.1113 | 0.405855 | 0.154537 | -0.0167 |
| 45 | Nicaragua | -0.31277 | -0.23147 | 0.232112 | 0.103423 | -0.19638 | 0.281213 | -0.02065 |
| 46 | Tajikistan | 0.032905 | -0.56481 | 0.298435 | -0.04933 | 0.286401 | -0.15013 | -0.02442 |
| 47 | Tuvalu | 0.150786 | 0.103685 | 0.691382 | 0.070817 | -0.58147 | -0.61998 | -0.0308 |
| 48 | Uganda | -0.15365 | -0.37509 | -0.4356 | -0.30694 | 0.581907 | 0.363349 | -0.05434 |
| 49 | Gambia, The | -0.45538 | -0.08629 | 0.017895 | 0.129778 | -0.15273 | -0.02504 | -0.0953 |
| 50 | Mali | -0.21218 | -0.23106 | -0.18255 | -0.29749 | 0.386897 | -0.04213 | -0.09642 |
| 51 | Micronesia, Fed. Sts. | -0.02808 | 0.044986 | 0.069799 | 0.316725 | -0.40417 | -0.66775 | -0.11141 |
| 52 | Zambia | -0.01398 | -0.35426 | -0.04731 | -0.07948 | 0.303056 | -0.55552 | -0.12458 |
| 53 | Sao Tome and Principe | -0.06513 | 0.416852 | -0.05536 | -0.01201 | -0.52065 | -0.6106 | -0.14115 |
| 54 | Mauritania | -0.31342 | -0.27998 | -0.04341 | -0.21894 | -0.17649 | 0.098707 | -0.15559 |
| 55 | Sierra Leone | -0.04923 | -0.3396 | -0.49023 | 0.11846 | -0.16617 | -0.20295 | -0.18829 |
| 56 | Cameroon | -0.33608 | -0.41874 | 0.10723 | -0.1589 | 0.101027 | -0.43662 | -0.19035 |
| 57 | Timor-Leste | -0.37282 | -0.21221 | 0.086283 | 0.432846 | -0.67408 | -0.46295 | -0.20049 |
| 58 | Zimbabwe | -0.16584 | -0.13868 | -0.16432 | -0.48678 | -0.46139 | 0.211109 | -0.20098 |
| 59 | Myanmar | -0.29059 | 0.075565 | 0.457526 | -0.17931 | -0.12093 | -1.15453 | -0.20205 |
| 60 | Liberia | -0.47768 | -0.14699 | -0.19402 | -0.23901 | -0.16844 | -0.24811 | -0.24571 |
| 61 | Marshall Islands | 0.127603 | -0.10766 | 0.089237 | 0.330901 | -0.69479 | -1.23307 | -0.24796 |
| 62 | Niger | -0.2654 | -0.63205 | -0.42542 | -0.7227 | 0.338601 | 0.190373 | -0.25276 |
| 63 | Afghanistan | -0.2297 | 0.081822 | -0.54753 | -0.00905 | -0.39105 | -0.56674 | -0.27704 |
| 64 | Mozambique | -0.07728 | 0.071048 | -0.54299 | -0.6898 | -0.31835 | -0.26513 | -0.30375 |
| 65 | Guinea | -0.53699 | -0.69755 | -0.02544 | -0.66646 | -0.01886 | 0.105032 | -0.30671 |
| 66 | Solomon Islands | -0.02331 | -0.19956 | -0.35161 | -0.35527 | -0.07189 | -0.84626 | -0.30798 |
| 67 | Papua New Guinea | -0.42733 | -0.20056 | -0.31732 | -0.26572 | -0.01698 | -0.69641 | -0.32072 |
| 68 | Madagascar | -0.24931 | -0.22504 | -0.53249 | -0.32918 | -0.29181 | -0.31105 | -0.32314 |
| 69 | Haiti | -0.27428 | 0.586672 | -0.24086 | -0.31125 | -0.82586 | -0.91451 | -0.33001 |
| 70 | Burundi | -0.29326 | -0.5261 | -0.58438 | -0.50933 | -0.63882 | 0.326913 | -0.37083 |
| 71 | Congo, Rep. | -0.42095 | -0.33139 | -0.21451 | -0.03004 | -0.70382 | -0.61605 | -0.38613 |
| 72 | Angola | -0.53496 | -0.03026 | -0.15295 | -0.56374 | -0.83363 | -0.69364 | -0.4682 |
| 73 | Central African Republic | -0.0446 | -0.32443 | -0.57165 | -0.08778 | -0.92302 | -1.06998 | -0.50358 |
| 74 | Guinea-Bissau | -0.5636 | -0.06699 | -0.4075 | -0.51317 | -0.75815 | -0.81315 | -0.52043 |
| 75 | Comoros | -0.57087 | -0.51273 | -0.33662 | -0.39159 | -0.58277 | -0.744 | -0.5231 |
| 76 | Chad | -0.40747 | -0.30302 | -0.84945 | -0.83105 | -0.62751 | -0.23418 | -0.54211 |
| 77 | Congo, Dem. Rep. | -0.41142 | -0.67921 | -0.73101 | -0.87923 | -0.57574 | 0.008977 | -0.54461 |
| 78 | Sudan | -0.42613 | -0.42575 | 0.009877 | -0.7036 | -1.09683 | -0.84189 | -0.58072 |
| 79 | Eritrea | -0.15135 | 0.052468 | -0.34355 | 0.024908 | -2.30063 | -0.84071 | -0.59314 |
| 80 | Yemen, Rep. | 0.161517 | -0.35635 | 0.024481 | -0.53569 | -2.06751 | -1.01354 | -0.63118 |
| 81 | Somalia | -0.51377 | -0.10877 | -0.76224 | -0.49105 | -2.24777 | -0.80263 | -0.82104 |
| 82 | South Sudan | -0.66031 | -0.50391 | -0.86225 | -0.32214 | -2.27191 | -1.80079 | -1.07022 |
