# Supplementary material for: Estimating a panel MSK dataset for comparative analyses of national absorptive capacity systems, economic growth, and development in low and middle income countries
Source: PLoS One. 2022 Oct 20;17(10):e0274402. doi: 10.1371/journal.pone.0274402 (PMC9584427; doi:10.1371/journal.pone.0274402)
Supplement: S4 Table — (DOCX) [file pone.0274402.s004.docx]

**Supporting Information**

**S8 Table. Pairwise correlations for incomplete (m=0) and complete datasets (m=25)**

Overall, the correlations for incomplete and complete datasets are similar, suggesting the reliability of the imputation results.

**S8A Tables. Technology capacity pairwise correlations incomplete (above) and complete (below)**

| Variables | (1) | (2) | (3) | (4) | (5) | (6) | (7) | (8) |
| --- | --- | --- | --- | --- | --- | --- | --- | --- |
| **(1) Sci & tech. articles** | 1.000 |  |  |  |  |  |  |  |
| **(2) Intellectual payments (mil)** | 0.981 | 1.000 |  |  |  |  |  |  |
| **(3) Voc. & tech. students (mil)** | 0.621 | 0.653 | 1.000 |  |  |  |  |  |
| **(4) R&D expend. % of GDP** | 0.605 | 0.549 | 0.385 | 1.000 |  |  |  |  |
| **(5) R&D researchers (per mil)** | -0.015 | -0.020 | 0.064 | 0.187 | 1.000 |  |  |  |
| **(6) R&D technicians (per mil)** | 0.074 | 0.071 | 0.050 | 0.244 | 0.439 | 1.000 |  |  |
| **(7) High-tech exports (mil)** | 0.041 | 0.061 | -0.034 | 0.170 | 0.183 | 0.013 | 1.000 |  |
| **(8) ECI (econ. complexity)** | 0.244 | 0.268 | 0.096 | 0.338 | 0.545 | 0.323 | 0.080 | 1.000 |
|  | | | | | | | | |

| Variables | (1) | (2) | (3) | (4) | (5) | (6) | (7) | (8) |
| --- | --- | --- | --- | --- | --- | --- | --- | --- |
| **(1) Sci & tech. articles** | 1.000 |  |  |  |  |  |  |  |
| **(2) Intellectual payments (mil)** | 0.968 | 1.000 |  |  |  |  |  |  |
| **(3) Voc. & tech. students (mil)** | 0.527 | 0.487 | 1.000 |  |  |  |  |  |
| **(4) R&D expend. % of GDP** | 0.346 | 0.307 | 0.264 | 1.000 |  |  |  |  |
| **(5) R&D researchers (per mil)** | -0.005 | -0.004 | 0.072 | 0.122 | 1.000 |  |  |  |
| **(6) R&D technicians (per mil)** | 0.148 | 0.115 | 0.046 | 0.258 | 0.097 | 1.000 |  |  |
| **(7) High-tech exports (mil)** | 0.024 | 0.033 | -0.049 | 0.169 | 0.052 | 0.123 | 1.000 |  |
| **(8) ECI (econ. complexity)** | 0.193 | 0.176 | 0.040 | 0.085 | 0.409 | 0.244 | 0.066 | 1.000 |
|  | | | | | | | | |

**S8B Tables. Financial capacity pairwise correlations incomplete (above) and complete (below)**

| Variables | (1) | (2) | (3) | (4) | (5) | (6) | (7) | (8) | (9) | (10) | (11) |
| --- | --- | --- | --- | --- | --- | --- | --- | --- | --- | --- | --- |
| **(1) Tax revenue (% of GDP)** | 1.000 |  |  |  |  |  |  |  |  |  |  |
| **(2) Business startup cost** | -0.207 | 1.000 |  |  |  |  |  |  |  |  |  |
| **(3) Domestic credit by banks** | 0.072 | -0.333 | 1.000 |  |  |  |  |  |  |  |  |
| **(4) Days to start business** | 0.127 | 0.382 | -0.134 | 1.000 |  |  |  |  |  |  |  |
| **(5) Days enforcing contract** | 0.025 | 0.055 | -0.132 | 0.165 | 1.000 |  |  |  |  |  |  |
| **(6) Days to register property** | -0.045 | 0.193 | -0.166 | 0.250 | 0.199 | 1.000 |  |  |  |  |  |
| **(7) Openness measure** | -0.006 | 0.077 | 0.518 | 0.552 | -0.426 | 0.071 | 1.000 |  |  |  |  |
| **(8) Days to electric meter** | -0.200 | 0.034 | -0.140 | -0.078 | 0.129 | 0.137 | -0.138 | 1.000 |  |  |  |
| **(9) Business density** | 0.278 | -0.193 | 0.390 | -0.119 | -0.155 | -0.306 | 0.186 | -0.123 | 1.000 |  |  |
| **(10) Financial accountholders** | 0.171 | -0.224 | 0.400 | -0.052 | 0.020 | -0.141 | 0.134 | -0.116 | 0.475 | 1.000 |  |
| **(11) Commercial banks** | 0.086 | -0.319 | 0.526 | -0.183 | -0.237 | -0.202 | 0.220 | -0.096 | 0.553 | 0.531 | 1.000 |
|  | | | | | | | | | | | |

| Variables | (1) | (2) | (3) | (4) | (5) | (6) | (7) | (8) | (9) | (10) | (11) |
| --- | --- | --- | --- | --- | --- | --- | --- | --- | --- | --- | --- |
| **(1) Tax revenue (% of GDP)** | 1.000 |  |  |  |  |  |  |  |  |  |  |
| **(2) Business startup cost** | -0.068 | 1.000 |  |  |  |  |  |  |  |  |  |
| **(3) Domestic credit by banks** | 0.167 | -0.270 | 1.000 |  |  |  |  |  |  |  |  |
| **(4) Days to start business** | 0.290 | 0.419 | -0.126 | 1.000 |  |  |  |  |  |  |  |
| **(5) Days enforcing contract** | 0.175 | 0.060 | -0.068 | 0.169 | 1.000 |  |  |  |  |  |  |
| **(6) Days to register property** | 0.074 | 0.189 | -0.173 | 0.235 | 0.197 | 1.000 |  |  |  |  |  |
| **(7) Openness measure** | 0.153 | 0.095 | 0.476 | 0.537 | -0.380 | 0.009 | 1.000 |  |  |  |  |
| **(8) Days to electric meter** | -0.131 | -0.104 | 0.082 | -0.122 | 0.081 | 0.038 | -0.081 | 1.000 |  |  |  |
| **(9) Business density** | 0.273 | -0.145 | 0.339 | -0.125 | -0.119 | -0.201 | 0.138 | 0.026 | 1.000 |  |  |
| **(10) Financial accountholders** | 0.199 | -0.231 | 0.451 | -0.068 | 0.053 | -0.138 | 0.111 | 0.094 | 0.401 | 1.000 |  |
| **(11) Commercial banks** | 0.043 | -0.260 | 0.501 | -0.175 | -0.216 | -0.123 | 0.218 | -0.006 | 0.455 | 0.533 | 1.000 |
|  | | | | | | | | | | | |

**S8C Tables. Human capacity pairwise correlations incomplete (above) and complete (below)**

| Variables | (1) | (2) | (3) | (4) | (5) | (6) | (7) | (8) | (9) | (10) |
| --- | --- | --- | --- | --- | --- | --- | --- | --- | --- | --- |
| **(1) Primary enrollment (gross)** | 1.000 |  |  |  |  |  |  |  |  |  |
| **(2) Sec. enrollment (gross)** | 0.178 | 1.000 |  |  |  |  |  |  |  |  |
| **(3) Primary pupil-teacher ratio** | 0.064 | -0.787 | 1.000 |  |  |  |  |  |  |  |
| **(4) Primary completion rate** | 0.370 | 0.867 | -0.694 | 1.000 |  |  |  |  |  |  |
| **(5) Govt. expend. on educ.** | 0.140 | 0.240 | -0.261 | 0.252 | 1.000 |  |  |  |  |  |
| **(6) Human Capital Index 0-1** | 0.052 | 0.908 | -0.717 | 0.792 | 0.164 | 1.000 |  |  |  |  |
| **(7) Advanced educ. labor** | 0.171 | -0.005 | 0.005 | 0.001 | -0.143 | 0.251 | 1.000 |  |  |  |
| **(8) Compulsory educ. (years)** | -0.288 | 0.338 | -0.260 | 0.171 | 0.234 | 0.364 | -0.126 | 1.000 |  |  |
| **(9) Industry employment** | -0.044 | 0.637 | -0.546 | 0.538 | 0.060 | 0.534 | 0.001 | 0.306 | 1.000 |  |
| **(10) Service employment** | -0.162 | 0.620 | -0.648 | 0.449 | 0.222 | 0.372 | -0.109 | 0.268 | 0.559 | 1.000 |
|  | | | | | | | | | | |

| Variables | (1) | (2) | (3) | (4) | (5) | (6) | (7) | (8) | (9) | (10) |
| --- | --- | --- | --- | --- | --- | --- | --- | --- | --- | --- |
| **(1) Primary enrollment (gross)** | 1.000 |  |  |  |  |  |  |  |  |  |
| **(2) Sec. enrollment (gross)** | 0.174 | 1.000 |  |  |  |  |  |  |  |  |
| **(3) Primary pupil-teacher ratio** | 0.020 | -0.708 | 1.000 |  |  |  |  |  |  |  |
| **(4) Primary completion rate** | 0.372 | 0.815 | -0.646 | 1.000 |  |  |  |  |  |  |
| **(5) Govt. expend. on educ.** | 0.107 | 0.325 | -0.284 | 0.346 | 1.000 |  |  |  |  |  |
| **(6) Human Capital Index 0-1** | 0.187 | 0.796 | -0.619 | 0.723 | 0.204 | 1.000 |  |  |  |  |
| **(7) Advanced educ. labor** | 0.011 | -0.129 | 0.181 | -0.144 | -0.067 | -0.034 | 1.000 |  |  |  |
| **(8) Compulsory educ. (years)** | -0.306 | 0.335 | -0.211 | 0.178 | 0.176 | 0.308 | -0.076 | 1.000 |  |  |
| **(9) Industry employment** | -0.024 | 0.633 | -0.529 | 0.514 | 0.147 | 0.494 | -0.174 | 0.345 | 1.000 |  |
| **(10) Service employment** | -0.105 | 0.623 | -0.641 | 0.446 | 0.263 | 0.472 | -0.163 | 0.313 | 0.565 | 1.000 |
|  | | | | | | | | | | |

**S8D Tables. Infrastructure capacity pairwise correlations incomplete (above) and complete (below)**

| Variables | (1) | (2) | (3) | (4) | (5) | (6) | (7) |
| --- | --- | --- | --- | --- | --- | --- | --- |
| **(1) Mobile subscriptions** | 1.000 |  |  |  |  |  |  |
| **(2) Access to electricity** | 0.514 | 1.000 |  |  |  |  |  |
| **(3) Broadband subscriptions** | 0.490 | 0.519 | 1.000 |  |  |  |  |
| **(4) Telephone subscriptions** | 0.343 | 0.682 | 0.694 | 1.000 |  |  |  |
| **(5) Energy use (per capita)** | 0.371 | 0.567 | 0.573 | 0.556 | 1.000 |  |  |
| **(6) Logistic perf. Index 1-5** | 0.344 | 0.250 | 0.244 | 0.160 | 0.154 | 1.000 |  |
| **(7) Internet users** | 0.680 | 0.651 | 0.733 | 0.579 | 0.580 | 0.343 | 1.000 |
|  | | | | | | | |

| Variables | (1) | (2) | (3) | (4) | (5) | (6) | (7) |
| --- | --- | --- | --- | --- | --- | --- | --- |
| **(1) Mobile subscriptions** | 1.000 |  |  |  |  |  |  |
| **(2) Access to electricity** | 0.509 | 1.000 |  |  |  |  |  |
| **(3) Broadband subscriptions** | 0.471 | 0.496 | 1.000 |  |  |  |  |
| **(4) Telephone subscriptions** | 0.342 | 0.664 | 0.684 | 1.000 |  |  |  |
| **(5) Energy use (per capita)** | 0.363 | 0.559 | 0.702 | 0.585 | 1.000 |  |  |
| **(6) Logistic perf. Index 1-5** | 0.238 | 0.261 | 0.100 | 0.092 | 0.115 | 1.000 |  |
| **(7) Internet users** | 0.669 | 0.643 | 0.732 | 0.571 | 0.592 | 0.240 | 1.000 |
|  | | | | | | | |

**S8E Tables. Public Policy capacity pairwise correlations incomplete (above) and complete (below)**

| Variables | (1) | (2) | (3) | (4) | (5) |
| --- | --- | --- | --- | --- | --- |
| **(1) CPIA econ. mgmt.** | 1.000 |  |  |  |  |
| **(2) Public sect. mgmt. & instit** | 0.612 | 1.000 |  |  |  |
| **(3) Structural policies** | 0.649 | 0.740 | 1.000 |  |  |
| **(4) Statistical capacity 0-100** | 0.498 | 0.437 | 0.527 | 1.000 |  |
| **(5) Legal Rights Index 0-12** | 0.218 | 0.189 | 0.293 | 0.067 | 1.000 |
|  | | | | | |

| Variables | (1) | (2) | (3) | (4) | (5) |
| --- | --- | --- | --- | --- | --- |
| **(1) CPIA econ. mgmt.** | 1.000 |  |  |  |  |
| **(2) Public sect. mgmt. & instit** | 0.625 | 1.000 |  |  |  |
| **(3) Structural policies** | 0.641 | 0.740 | 1.000 |  |  |
| **(4) Statistical capacity 0-100** | 0.518 | 0.493 | 0.558 | 1.000 |  |
| **(5) Legal Rights Index 0-12** | 0.182 | 0.274 | 0.337 | 0.160 | 1.000 |
|  | | | | | |

**S8F Tables. Social capacity pairwise correlations incomplete (above) and complete (below)**

| Variables | (1) | (2) | (3) | (4) | (5) | (6) |
| --- | --- | --- | --- | --- | --- | --- |
| **(1) Human resources rating** | 1.000 |  |  |  |  |  |
| **(2) Equity of public resc use** | 0.620 | 1.000 |  |  |  |  |
| **(3) Social protection rating** | 0.627 | 0.655 | 1.000 |  |  |  |
| **(4) Social inclusion o..** | 0.852 | 0.827 | 0.815 | 1.000 |  |  |
| **(5) National headcount poverty** | -0.387 | -0.244 | -0.374 | -0.410 | 1.000 |  |
| **(6) Social contributions** | 0.213 | 0.169 | 0.326 | 0.338 | -0.187 | 1.000 |
|  | | | | | | |

| Variables | (1) | (2) | (3) | (4) | (5) | (6) |
| --- | --- | --- | --- | --- | --- | --- |
| **(1) Human resources rating** | 1.000 |  |  |  |  |  |
| **(2) Equity of public resc use** | 0.640 | 1.000 |  |  |  |  |
| **(3) Social protection rating** | 0.644 | 0.658 | 1.000 |  |  |  |
| **(4) Social inclusion o..** | 0.865 | 0.832 | 0.819 | 1.000 |  |  |
| **(5) National headcount poverty** | -0.395 | -0.225 | -0.303 | -0.364 | 1.000 |  |
| **(6) Social contributions** | 0.217 | 0.131 | 0.290 | 0.305 | -0.156 | 1.000 |
|  | | | | | | |
