# Supplementary material for: Estimating a panel MSK dataset for comparative analyses of national absorptive capacity systems, economic growth, and development in low and middle income countries
Source: PLoS One. 2022 Oct 20;17(10):e0274402. doi: 10.1371/journal.pone.0274402 (PMC9584427; doi:10.1371/journal.pone.0274402)
Supplement: S1 Fig — (DOCX) [file pone.0274402.s005.docx]

**Supporting Information**

**S3 Fig. Construction of the MSK Dataset**
