# Supplementary material for: Estimating a panel MSK dataset for comparative analyses of national absorptive capacity systems, economic growth, and development in low and middle income countries
Source: PLoS One. 2022 Oct 20;17(10):e0274402. doi: 10.1371/journal.pone.0274402 (PMC9584427; doi:10.1371/journal.pone.0274402)

**Supporting Information**

**S4 Fig. Kernel Densities for Select variables of Interest at Different Points**

Kernel densities are observed to examine the distribution pattern of select variables under each capacity at three periods (2005, 2010, and 2019). Overall technological capacity does not show any change in distribution, whereas infrastructure and social capacity show a rightward and leftward shift, respectively. The remaining three financial, human, and public policy capacities do not display any clear cross-country distributions’ evolution.

**S4A Fig. Technology Capacity:**


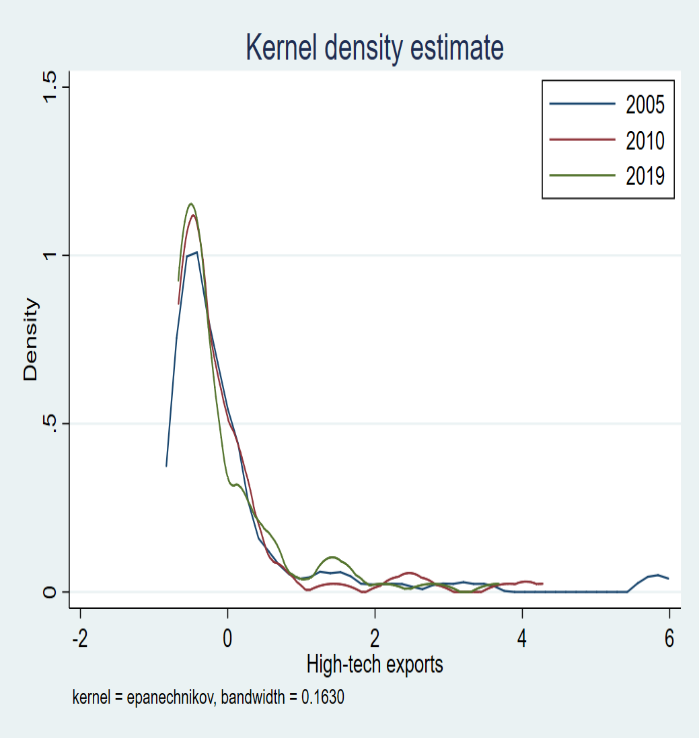

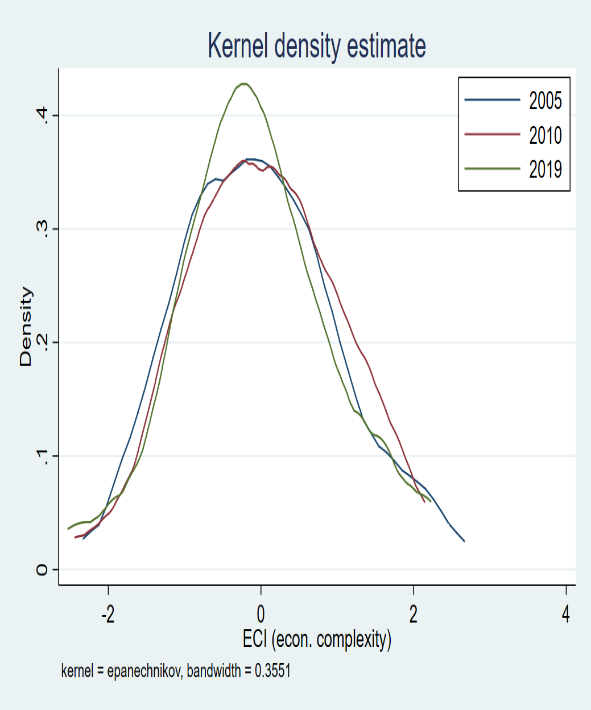


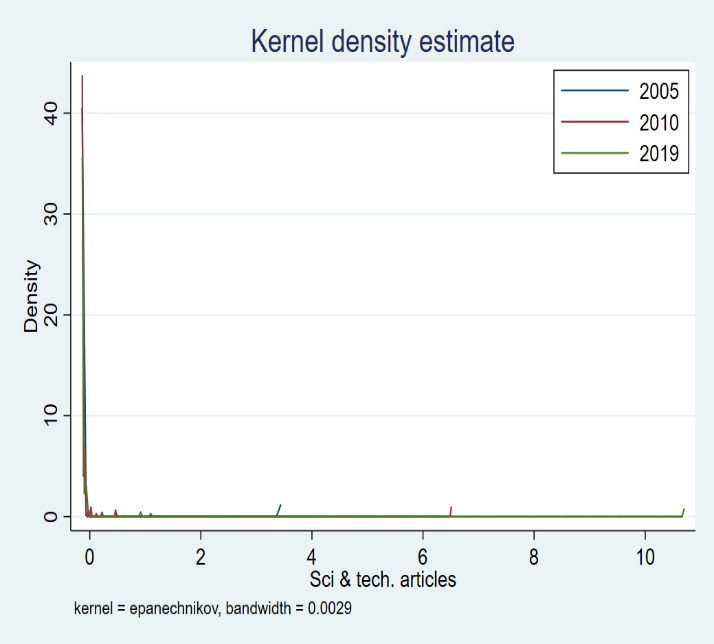


**S4B Fig. Financial Capacity**


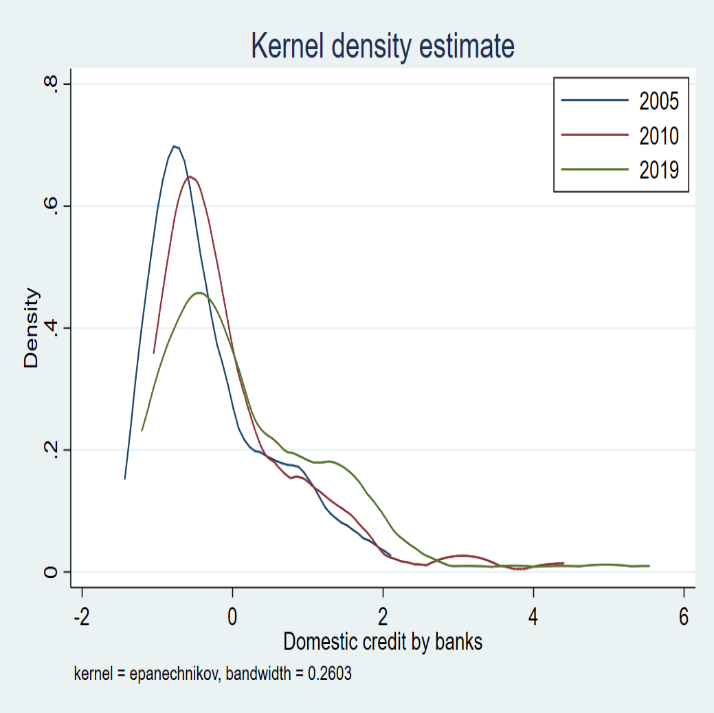

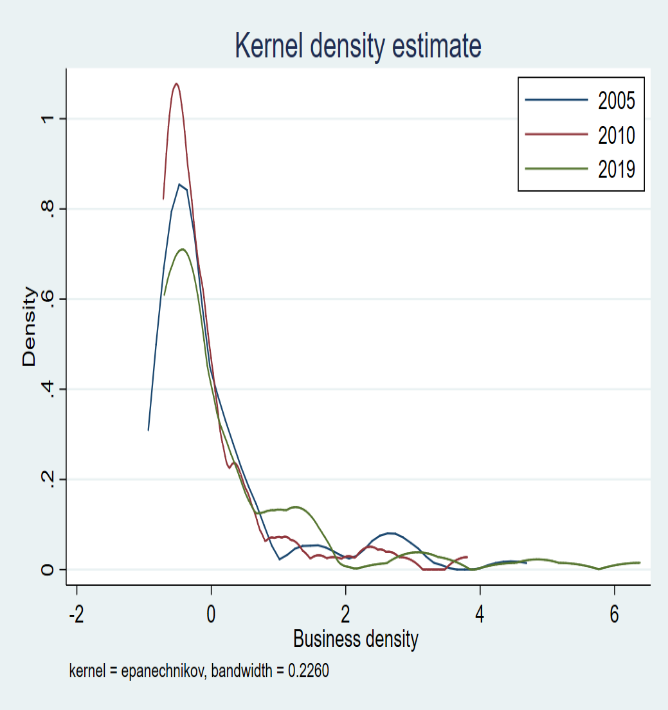


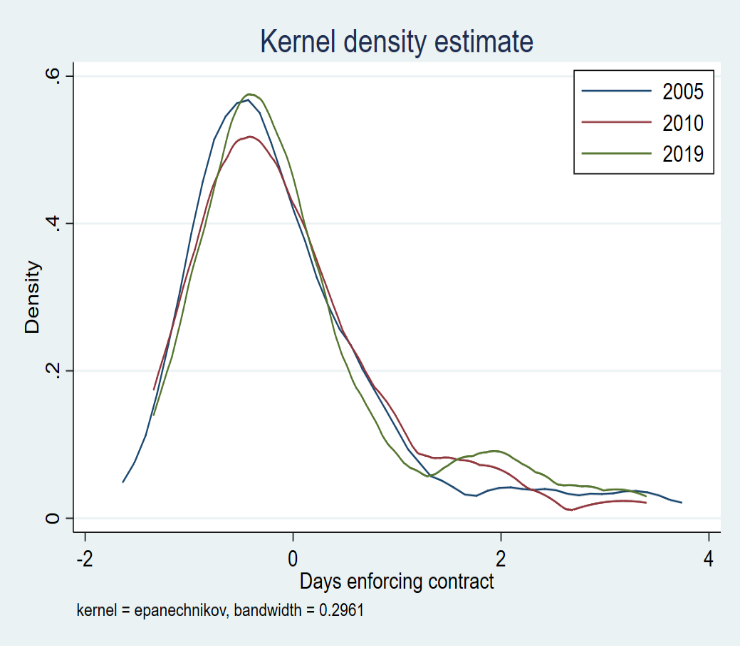


**S4C Fig. Human Capacity**


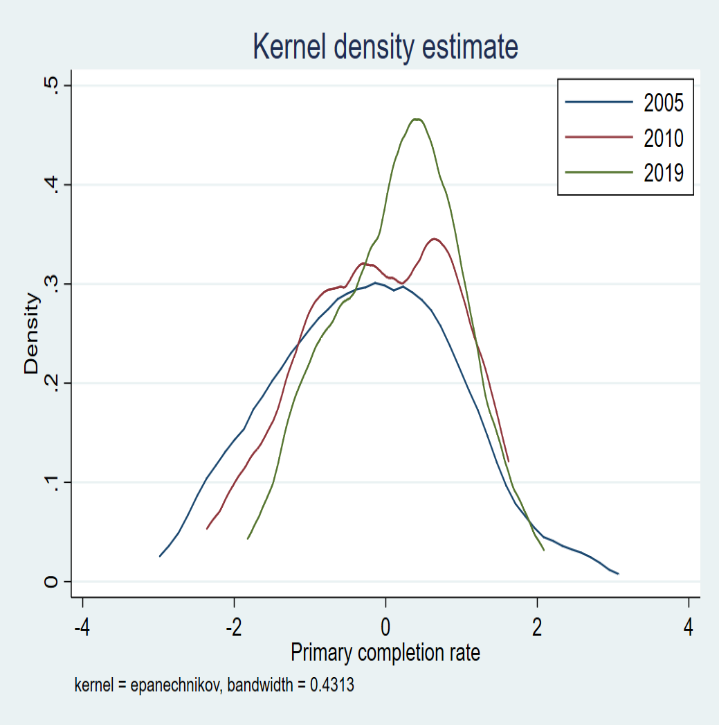

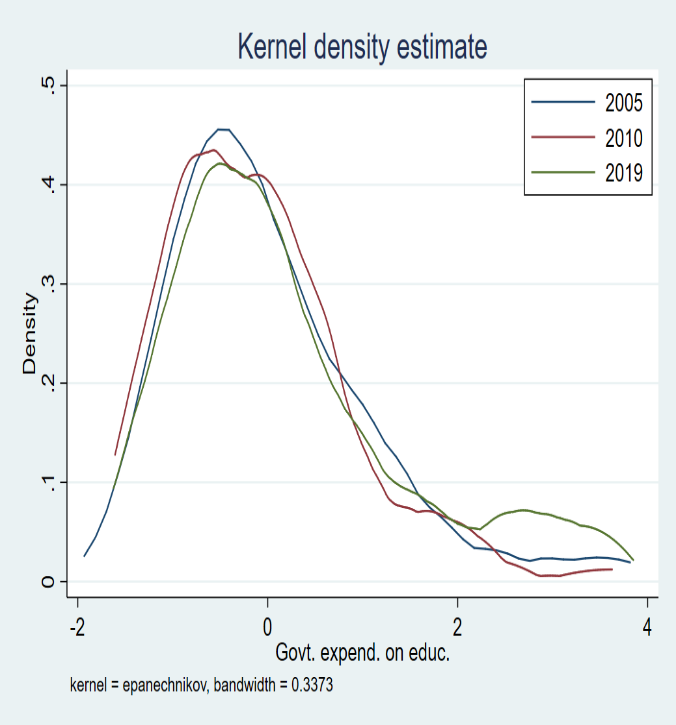


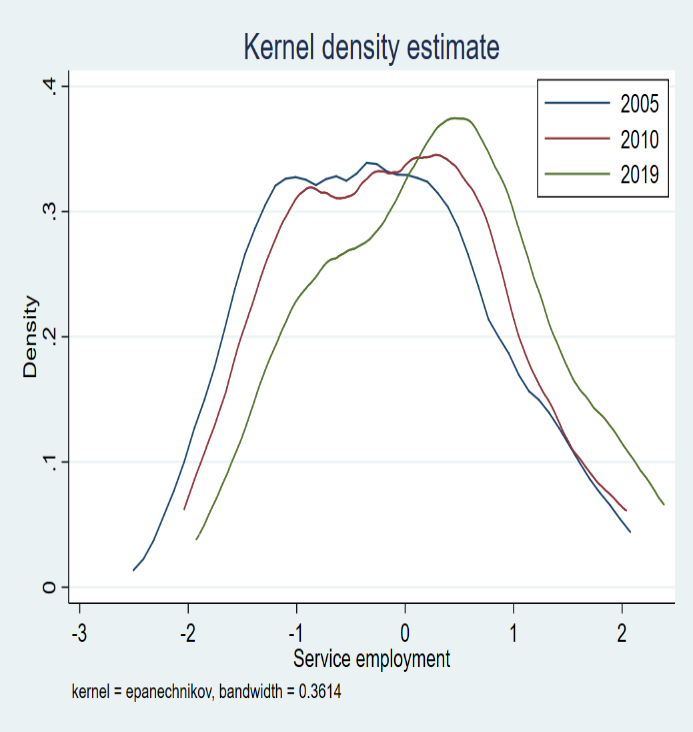


**S4D Fig. Infrastructure Capacity**


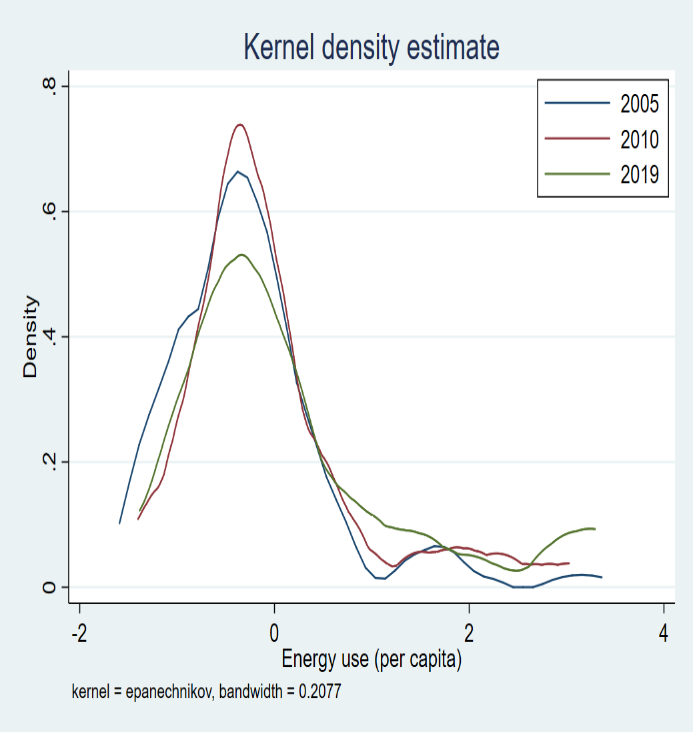

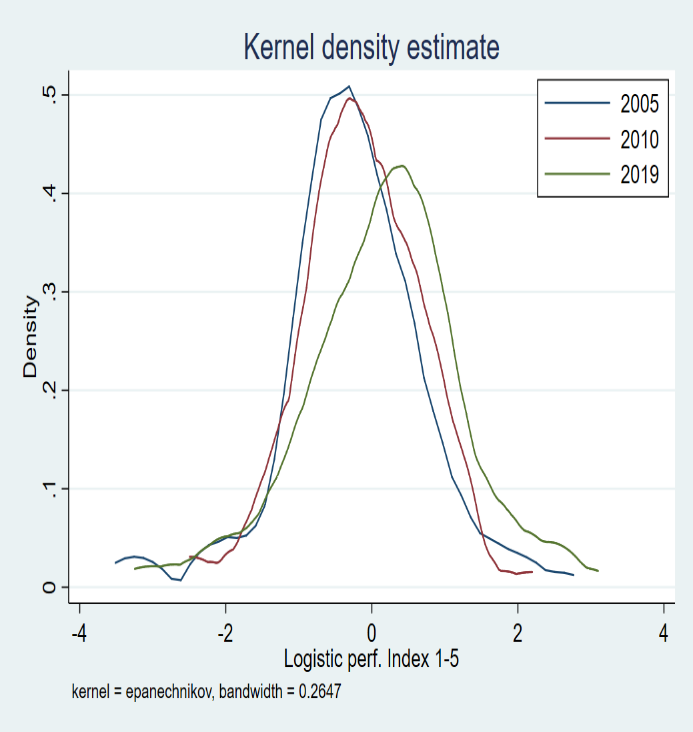


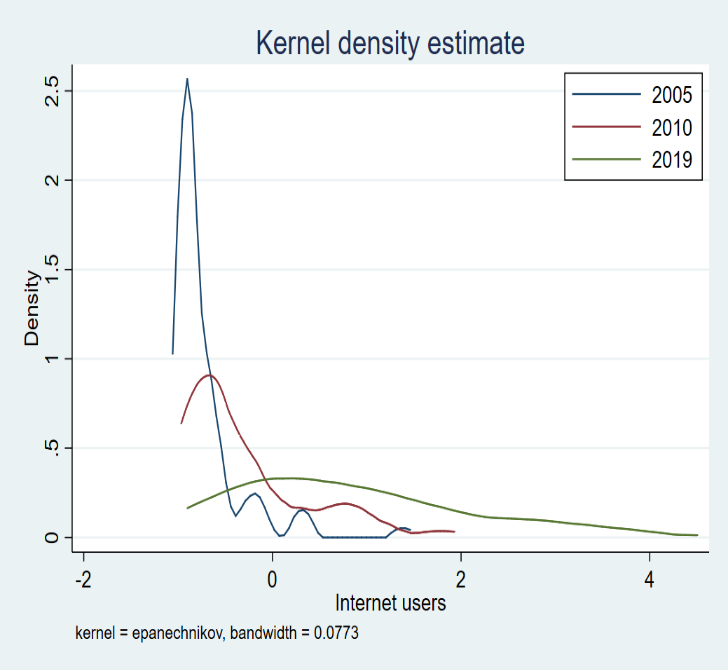

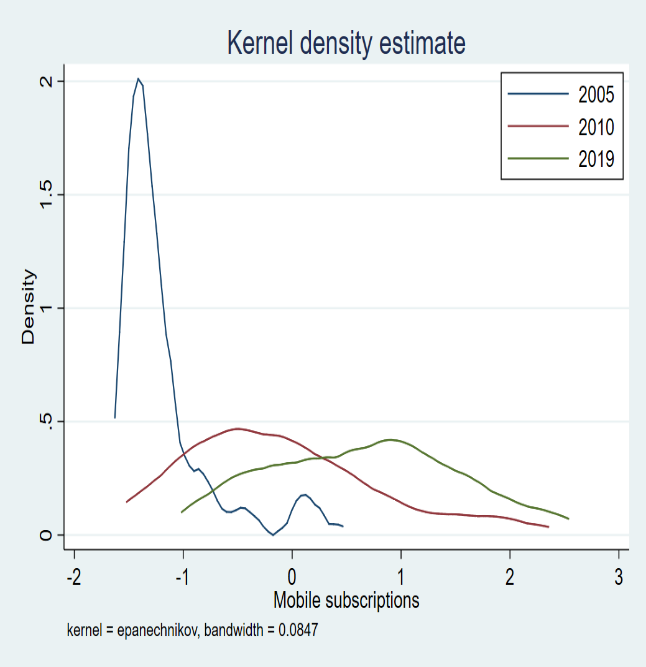


**S4E Fig. Public Policy Capacity**


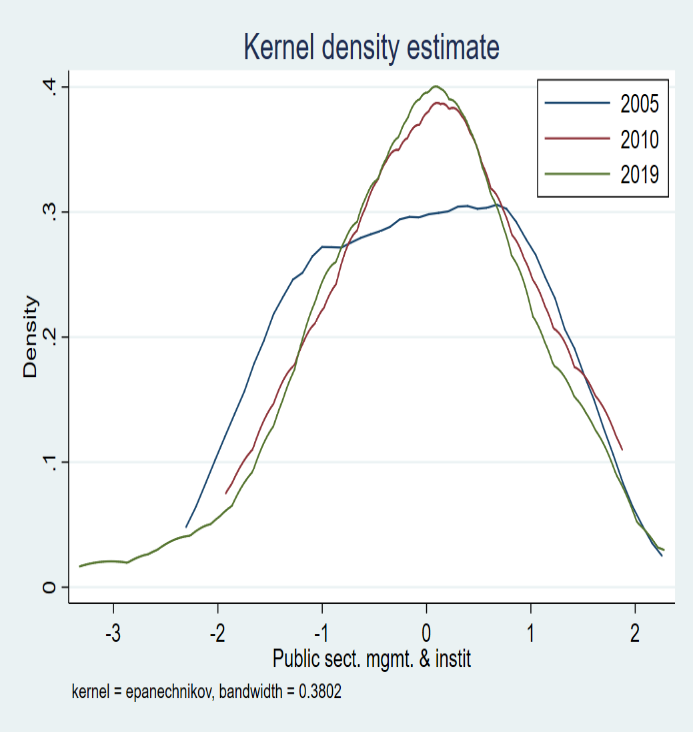

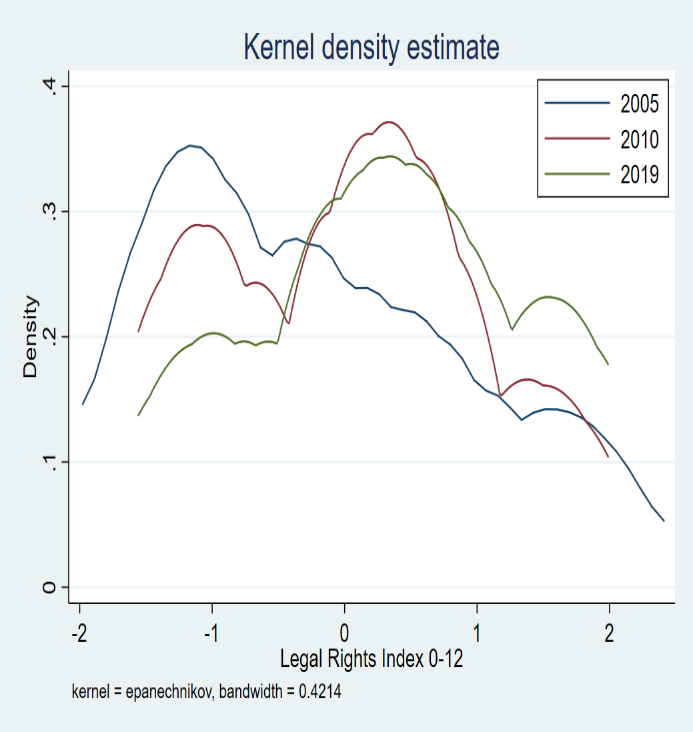


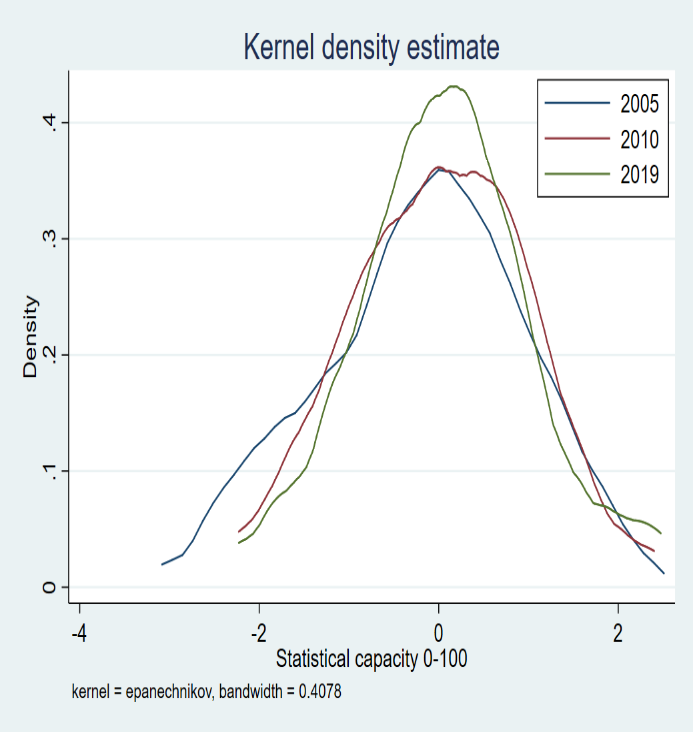


**S4F Fig. Social Capacity**


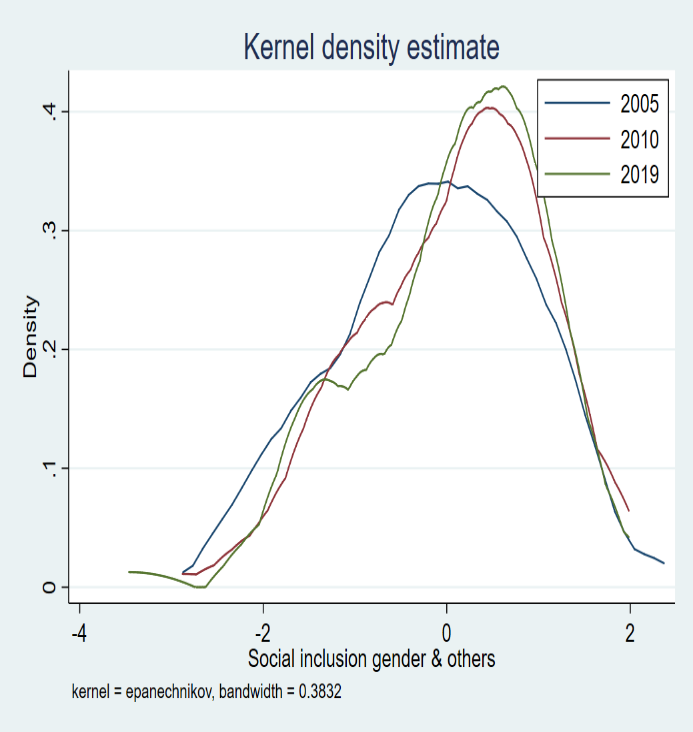

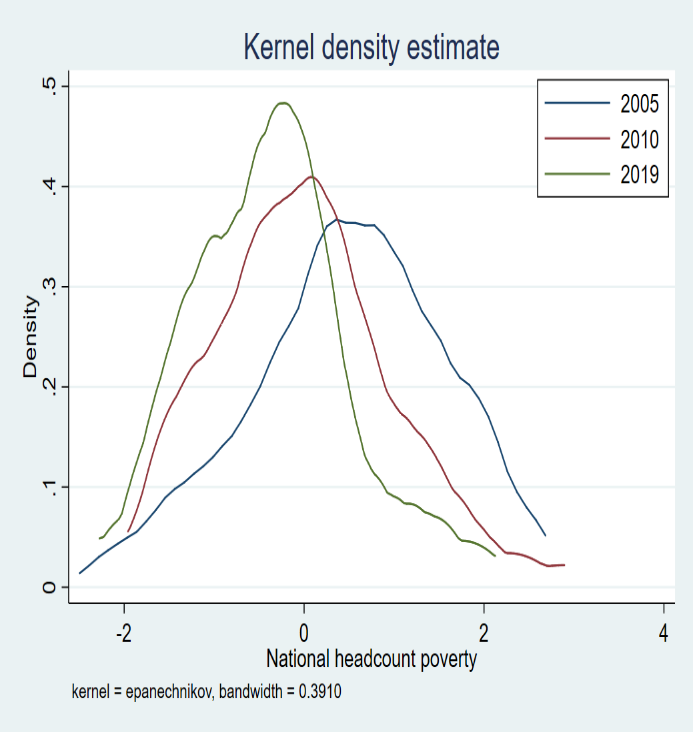


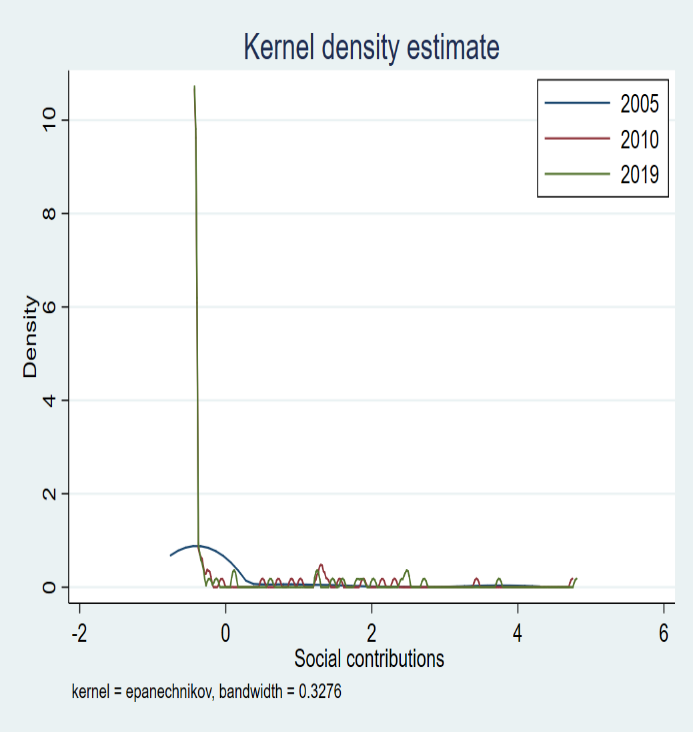

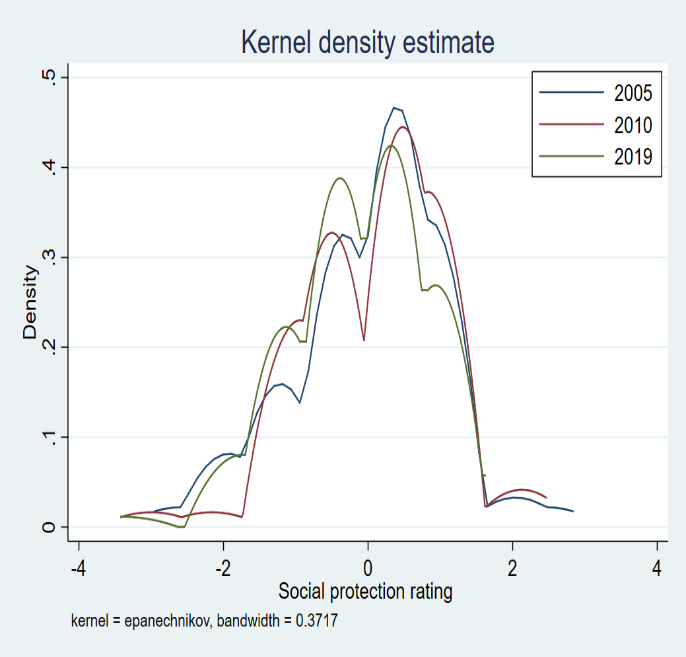

Supplement: S2 Fig — (DOCX) [file pone.0274402.s006.docx]
