# Supplementary material for: Estimating a panel MSK dataset for comparative analyses of national absorptive capacity systems, economic growth, and development in low and middle income countries
Source: PLoS One. 2022 Oct 20;17(10):e0274402. doi: 10.1371/journal.pone.0274402 (PMC9584427; doi:10.1371/journal.pone.0274402)

**Supporting Information**

**S5 Fig. Time Trends for Select Countries for Select Variables**

Trends of select variables under each capacity for select countries are given below. While some variables return a uniform trend, others indicate completely erratic or rising trends. The x-axis indicates the period from 2005 to 2019, whereas the y-axis shows the name of the variables.

**S5A Fig. Technology Capacity**


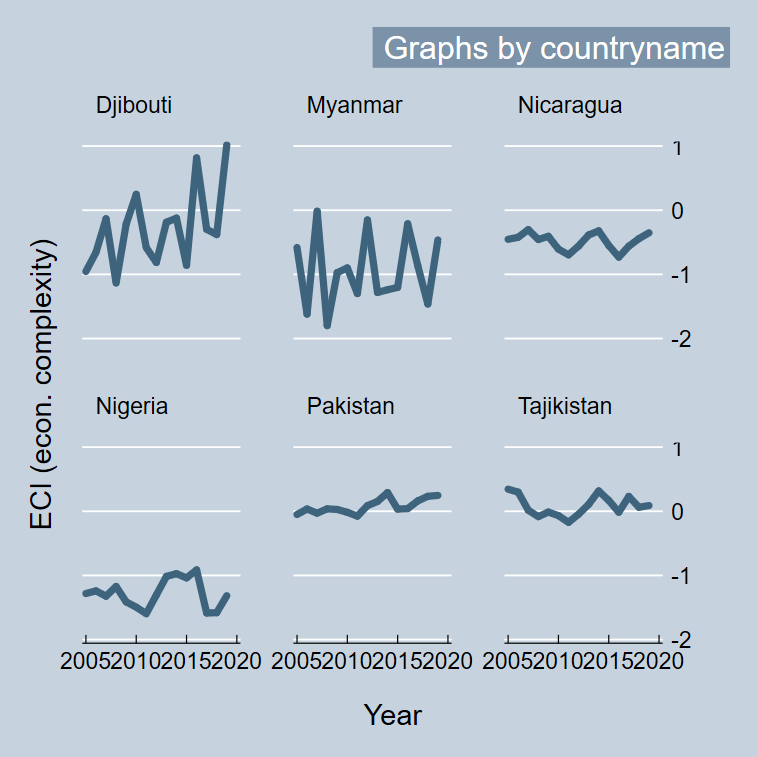

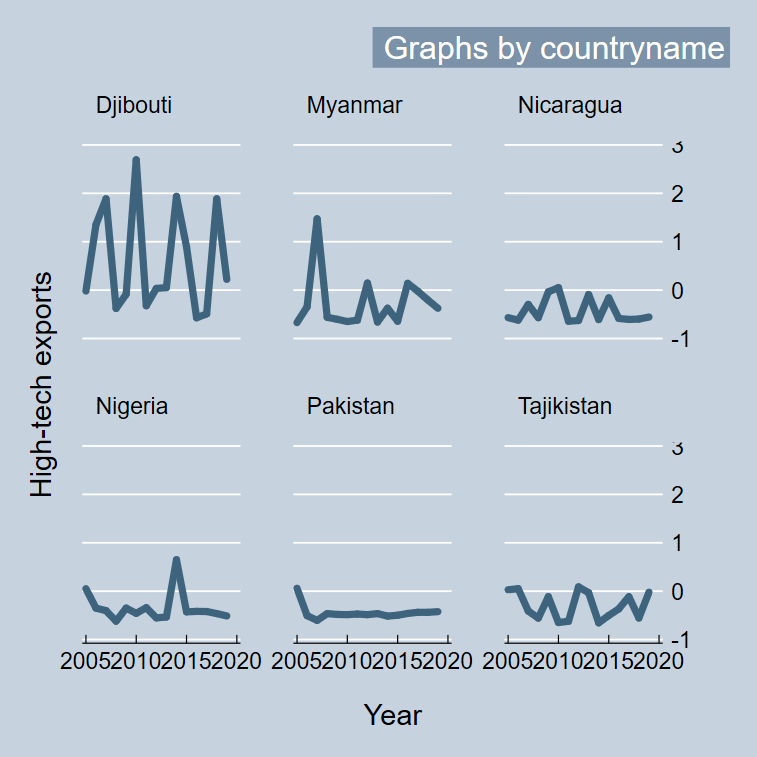


**S5A Fig. Technology Capacity (continued)**


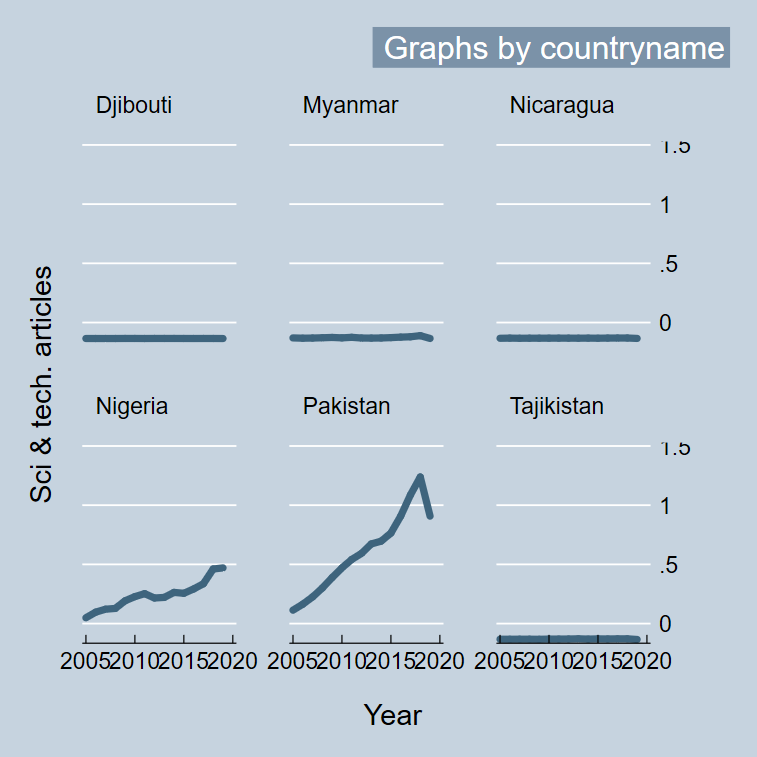


**S5B Fig. Financial Capacity**


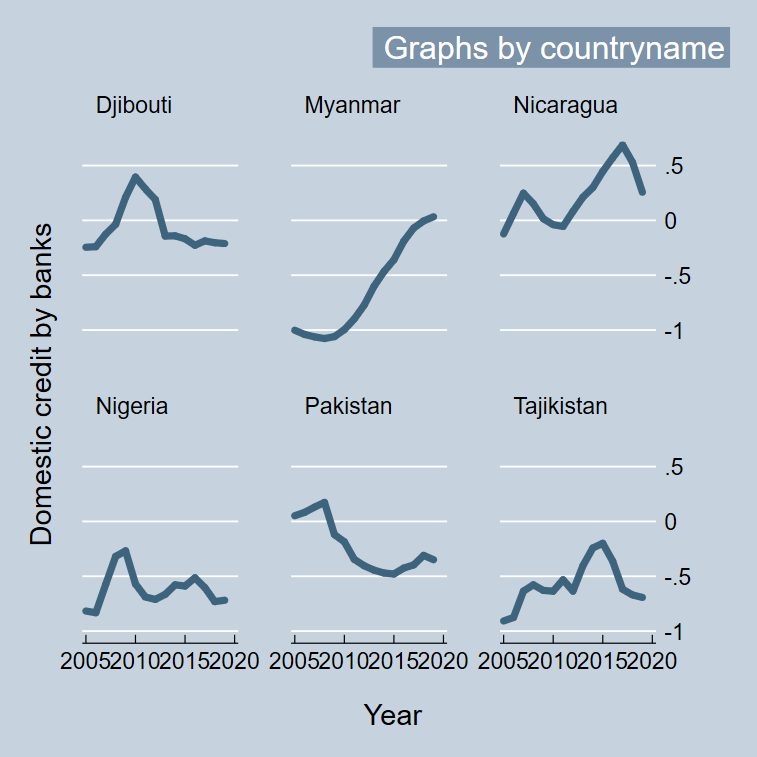

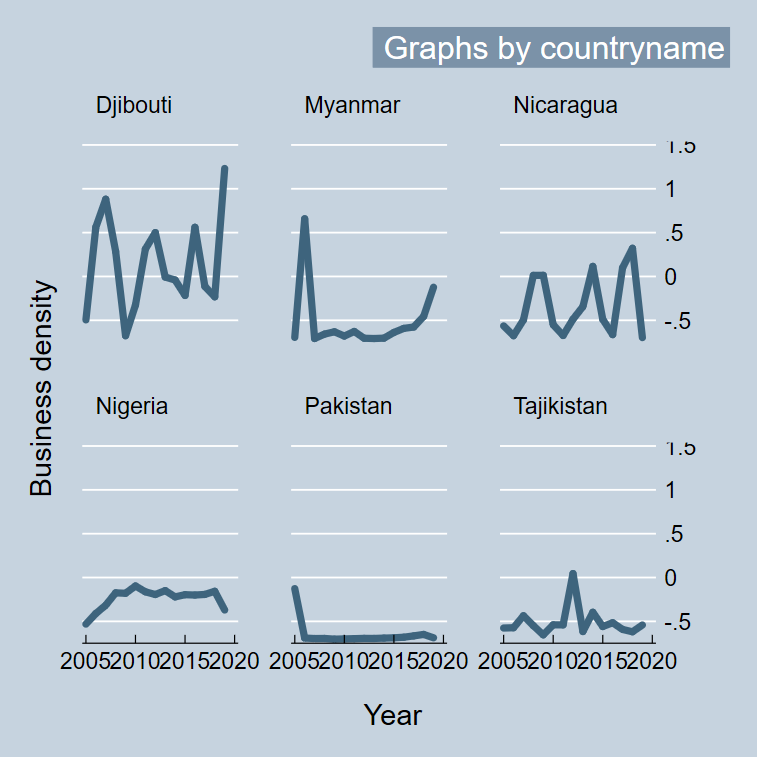


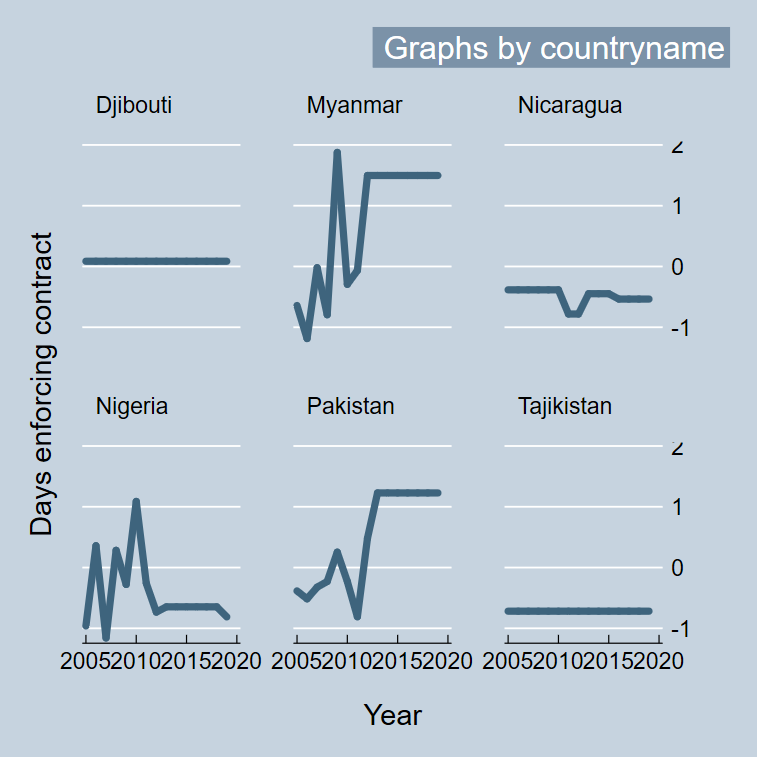


**S5C Fig. Human Capacity**


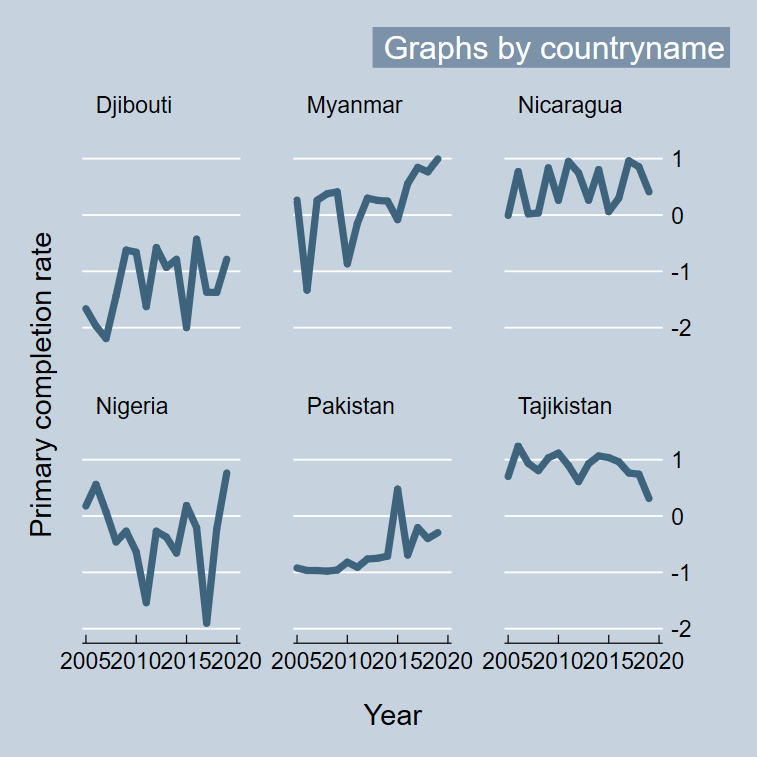

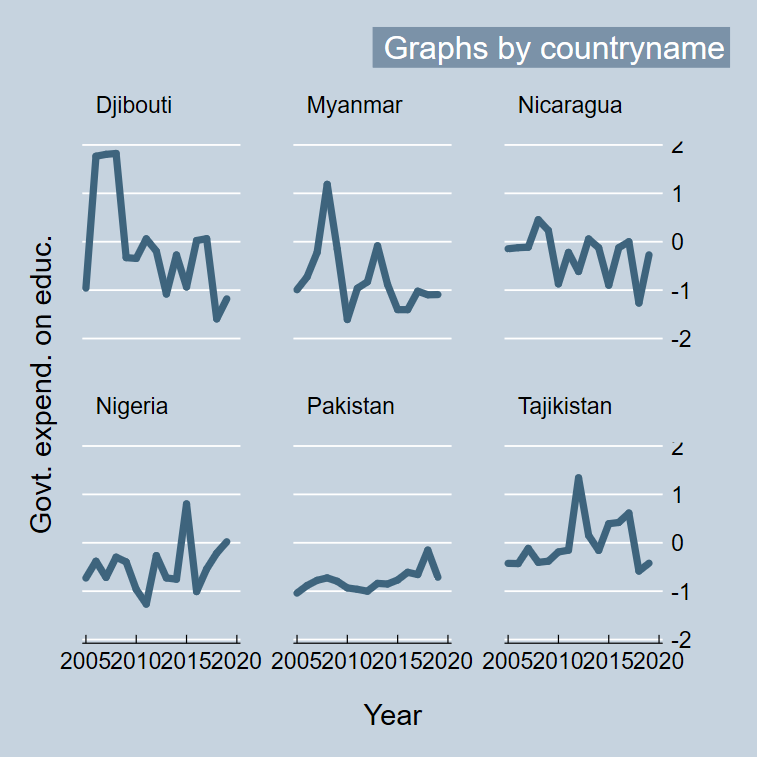


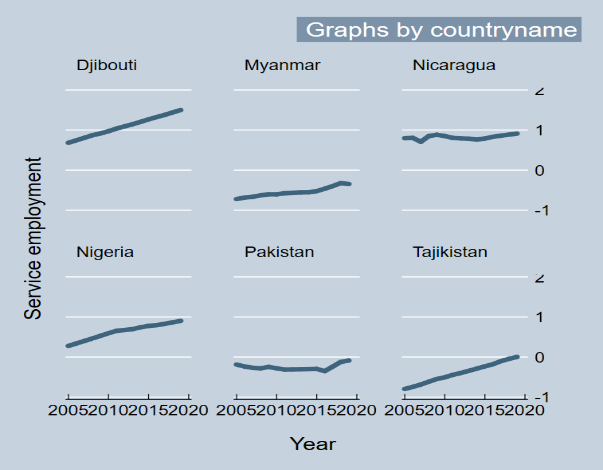

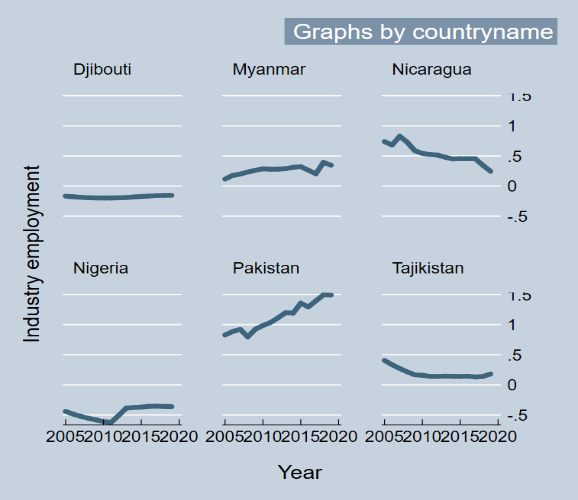


**S5D Fig. Infrastructure Capacity**


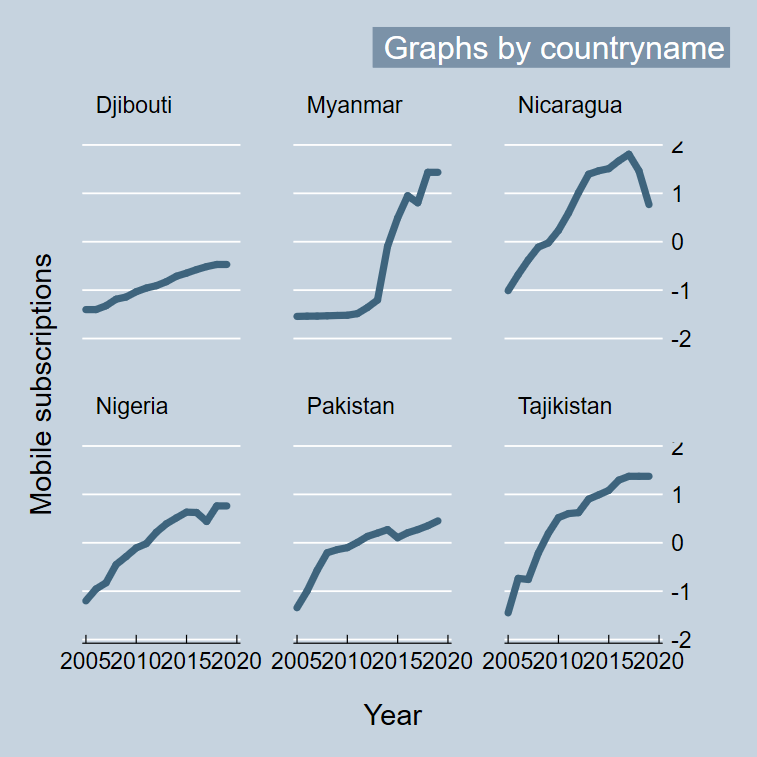

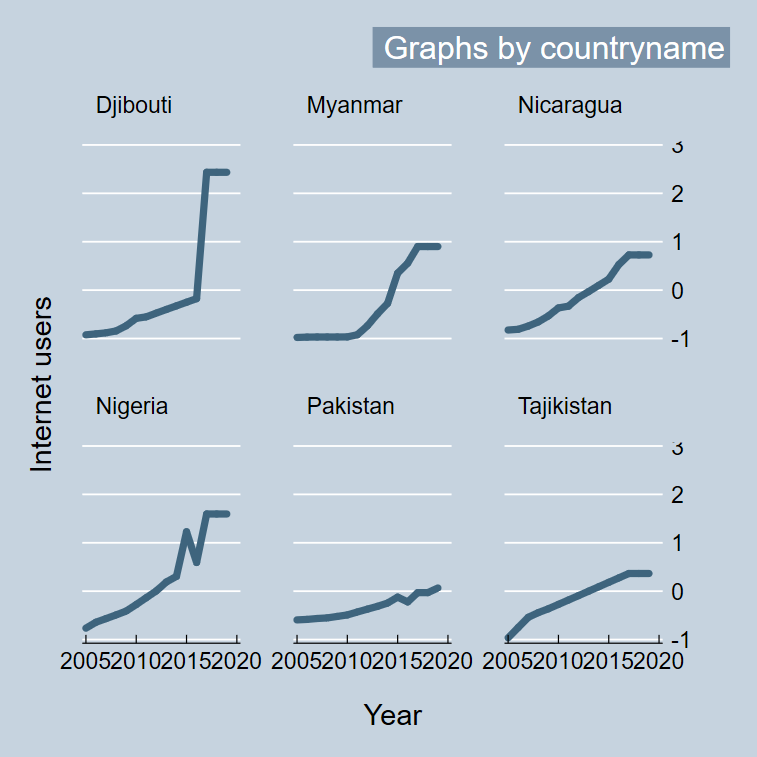


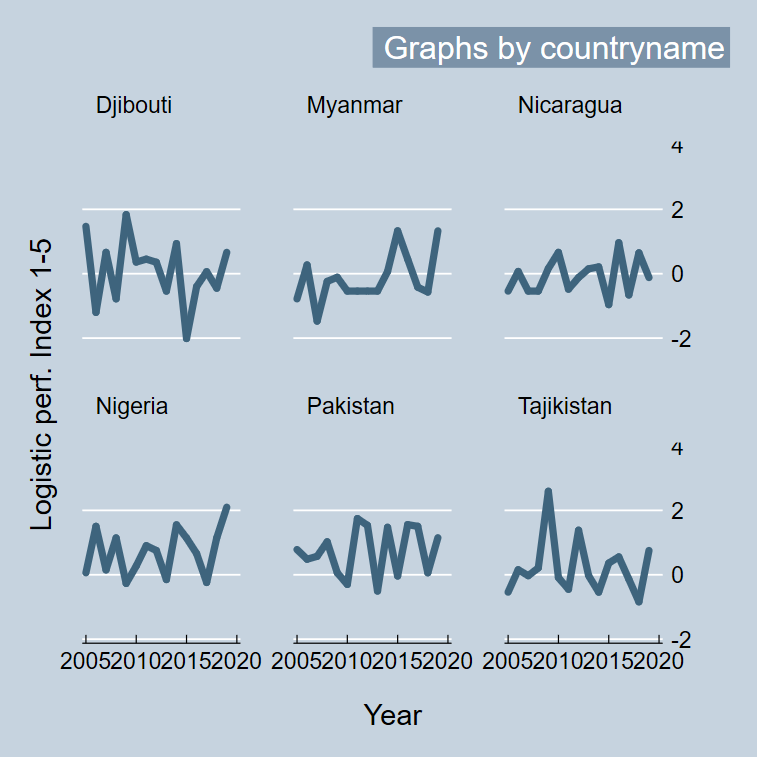


**S5E Fig. Public Policy Capacity**


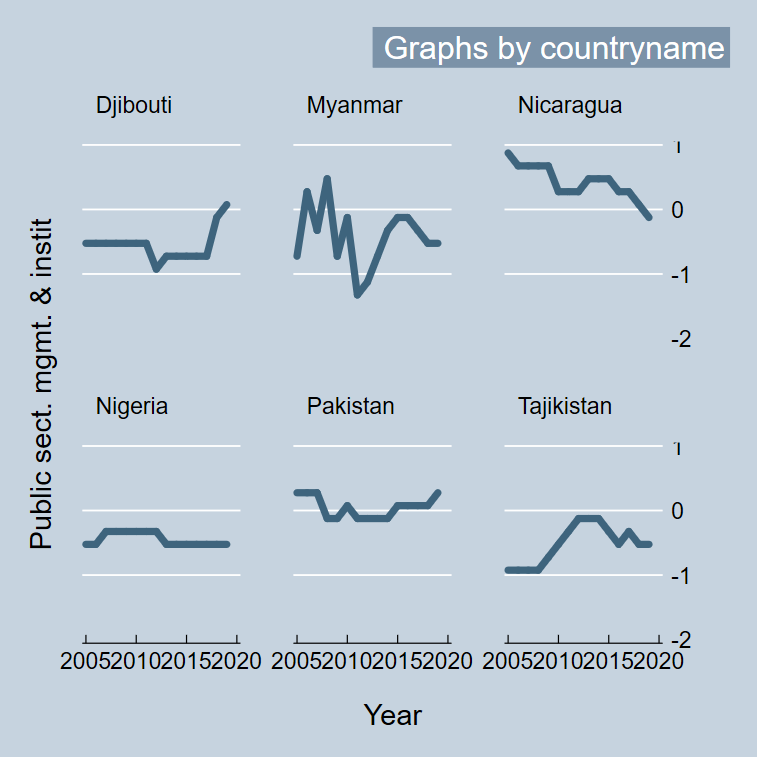

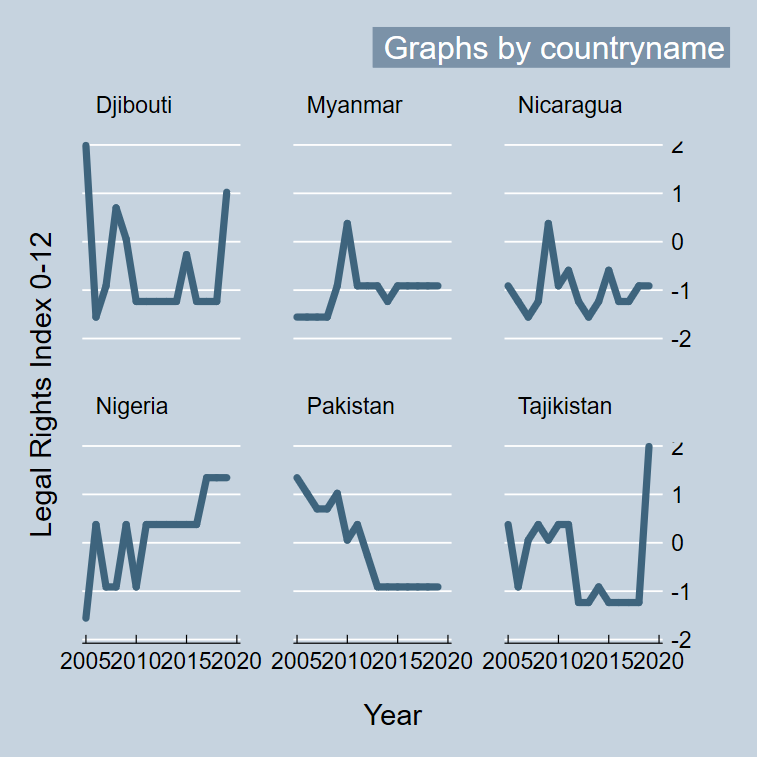


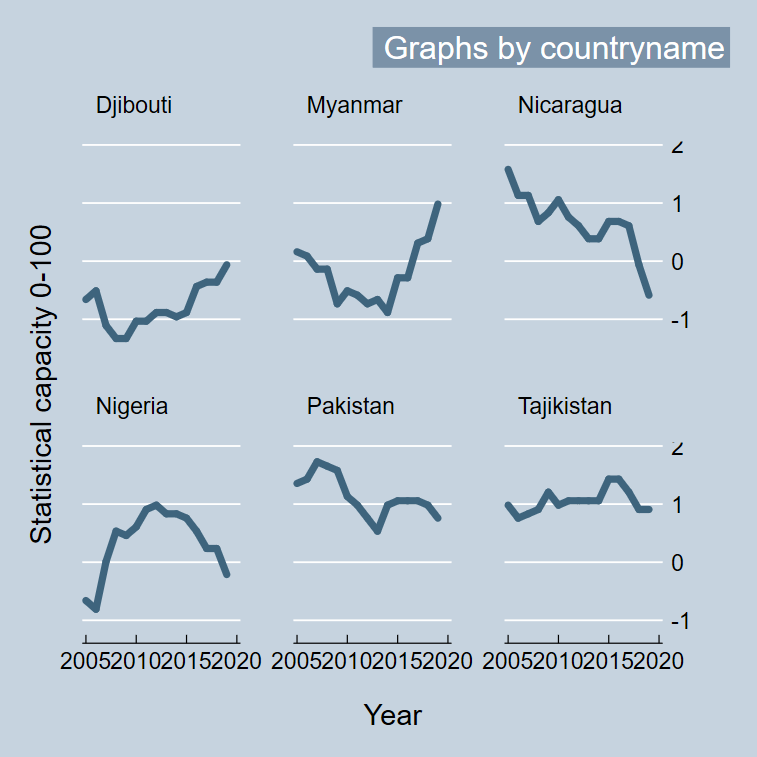

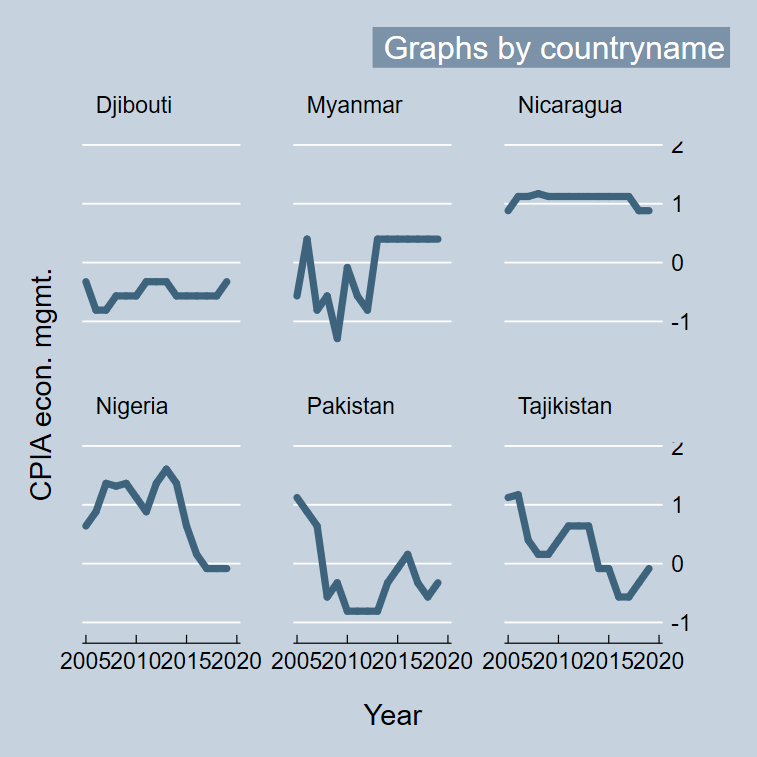


**S5F Fig. Social Capacity**


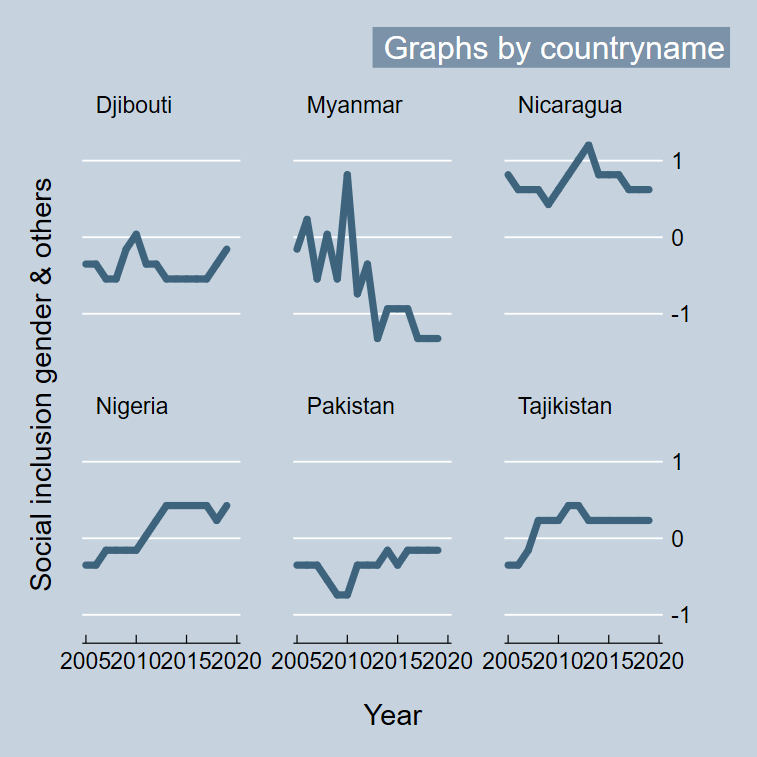

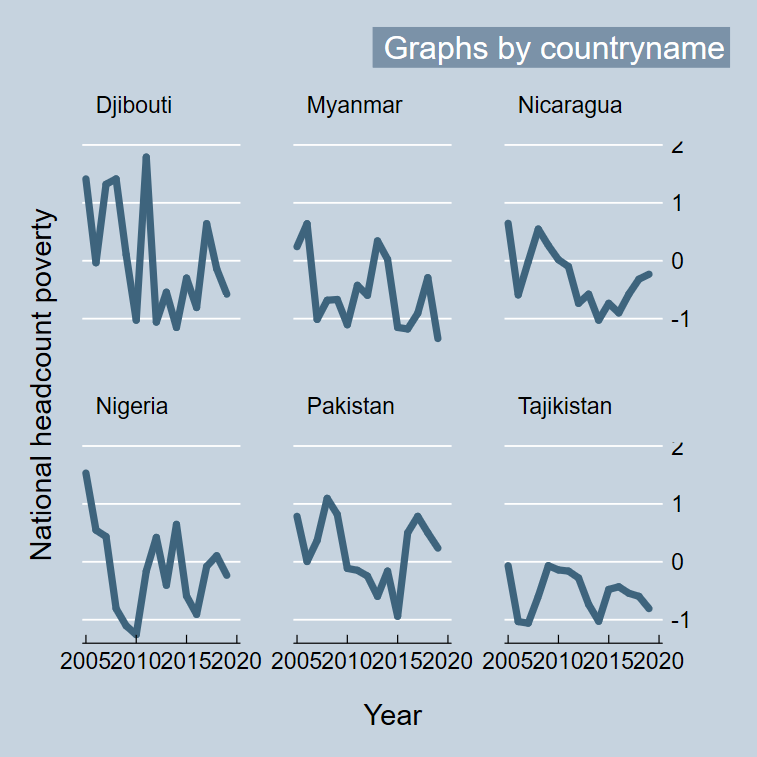


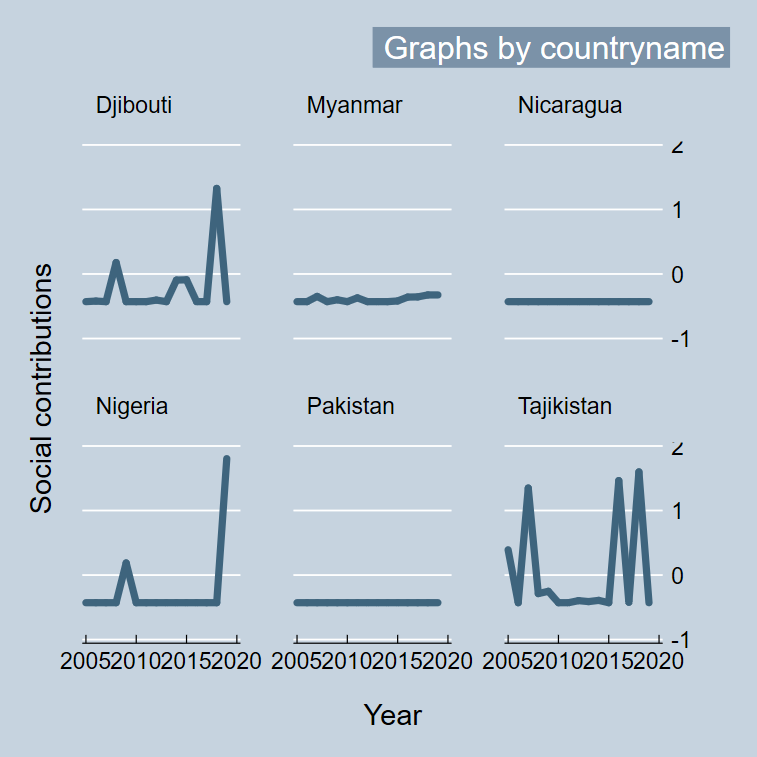

Supplement: S3 Fig — (DOCX) [file pone.0274402.s007.docx]
