# Supplementary material for: Estimating a panel MSK dataset for comparative analyses of national absorptive capacity systems, economic growth, and development in low and middle income countries
Source: PLoS One. 2022 Oct 20;17(10):e0274402. doi: 10.1371/journal.pone.0274402 (PMC9584427; doi:10.1371/journal.pone.0274402)
Supplement: S4 Fig — (DOCX) [file pone.0274402.s008.docx]

**Supporting Information**

**S7 Fig. Kernel Densities of the Observed and Complete Dataset**

Statistical distributions of observed and complete datasets are compared to examine how best the complete dataset represents the observed dataset. Distributions overall match, indicating the accuracy and reliability of imputation.

**S7A Fig. Technology capacity**

**S7B Fig. Financial Capacity**

**S7B Fig. Financial Capacity (continued)**

**S7C Fig. Human Capacity**

**S7C Fig. Human Capacity (Continued)**

**S7D Fig. Infrastructure Capacity**

**S7E Fig. Public Policy Capacity**

**S7F Fig. Social Capacity**
