# Supplementary material for: Estimating a panel MSK dataset for comparative analyses of national absorptive capacity systems, economic growth, and development in low and middle income countries
Source: PLoS One. 2022 Oct 20;17(10):e0274402. doi: 10.1371/journal.pone.0274402 (PMC9584427; doi:10.1371/journal.pone.0274402)
Supplement: S5 Fig — (DOCX) [file pone.0274402.s009.docx]

**Supporting Information**

**S9 Fig.** **Checking for Convergence through Trace Plots**

Trace plots show the convergence pattern of iterations involved in the imputation process. In this case, we see a healthy convergence. In other words, after plotting the mean and variance of the imputed values of different missing variables against the iteration number, the plots for imputed datasets freely intermingle without showing any definite trend. This suggests that the imputed dataset is of good quality.
