# Supplementary material for: Estimating a panel MSK dataset for comparative analyses of national absorptive capacity systems, economic growth, and development in low and middle income countries
Source: PLoS One. 2022 Oct 20;17(10):e0274402. doi: 10.1371/journal.pone.0274402 (PMC9584427; doi:10.1371/journal.pone.0274402)
Supplement: Attachment — Submitted filename: Response to reviewers.docx [file pone.0274402.s011.docx]

**Response to Reviewers: Estimating a new panel MSK dataset for comparative analyses of national absorptive capacity systems, economic growth, and development in low and middle income economies**

**Here I respond to reviewers and the editor’s suggestions/comments/concerns (green font). My response is in bold font.**

**Reviewer 1: (Key: Green text- Reviewer’s comment; Black text-Author’s response)**

**Just to clarify on the first footnote…when you are talking about relative poverty, the first dollar threshold that you mention is the world poverty threshold ($1,185 for 2021). Is that correct? And I am also assuming that is U.S. dollars.**

**That’s correct. I have included US dollars in the footnote.**

**Just a small issue, but on page 3 you mention NIS and NACS as being “fields”. My guess is that they should more correctly be referred to as organizations.**
**Thank you for pointing this out. By fields I meant the area of literature. I have rephrased the sentence for clarification purpose mentioning “innovation system and absorptive capacity literature”**

**On page 3, you mention “state-of-the-art statistical methods to address the missing data problem”. You can probably elaborate a little further here as to what these authors used. I am guessing that some may be different from the predictive mean matching (PMM) technique that you use.
That’s right. PMM is a univariate regression modeling technique where each single incomplete variable (outcome variable) is imputed using complete variables (predictors) in a series of regression models. Other classical versions within MI (such as MVN or multivariate normal MI) uses multivariate regression modeling where all the incomplete variables (outcome variables) are imputed using complete variables (predictors) in one step. I have mentioned this in the paragraph now:**

***MI uses state-of-the-art statistical methods to address the missing data problem (Rubin 1996; Enders 2017b). By treating missing variables as outcomes and complete variables as predictors, such statistical methods either impute all incomplete variables in a single computation step (multivariate regression model) or impute one variable at a time in a series (univariate regression models).***

**More explanation on several versions of MI is found in subsequent sections 3 and 4.**

**Your first reference to the MSK data set occurs on page 4 of the document. I believe that this is the proposed data set which you are building. Perhaps you can introduce it by calling it the “proposed” MSK data set.**

**Thank you for mentioning this. I added “proposed” before it.**

**Check out the second paragraph, sentence 3 on page 4. You actually refer to it as M1 rather than MI.**

**Thank you for catching this. I fixed it.**

**On page 4, you mention that “Public Policy and Social Capacity are operationalized very differently”. The reader may be curious as to how that is. You might want to consider elaborating on that.**

**I have further elaborated on this. The paragraph now reads as follows:**

***Secondly, though the MSK dataset employs some of the CANA dataset variables, it has an entirely different functional and operational conception of the capacities and the variables used to operationalize those capacities. Particularly, Public Policy and Social Capacity are operationalized very differently. In the MSK dataset, the Public Policy Capacity includes variables about public sector management and institutions, economic management, structural policies, the strength of legal rights, and statistical capacity scores of countries, whereas the Social Capacity includes variables on policies for social inclusion, human resource rating, social protection rating, equity of public resource use, poverty headcount ratio, and social contributions. On the other hand, in the CANA dataset, the analogous dimensions are the Political-institutional dimension (which comprises freedom of press and speech, human rights, women’s rights, and political rights, among other factors) and the Social capital dimension (which includes the importance of friends, family, marriage, trust, happiness, and Gini Index).***

**On page 5, you mention that “The incomplete (original or observed) dataset is constructed from reputable data sources and contains many missing values.” Some elaboration on these data sources might be necessary here.**

**I have included the data sources in footnote 3. Also, complete details about data sources are available in Supporting Information S2 Table, which I indicated in the text now. The footnote 3 reads as:**

***The incomplete dataset was compiled from publicly available data from the World Bank, International Monetary Fund (IMF), International Labor Organization (ILO), United Nations COMTRADE, United Nations Educational, Scientific and Cultural Organization (UNESCO), among others.***

**You mention the word “missingness” throughout the document. I am assuming that is a commonly used term in the imputation world.**

**Yes, that’s correct.**

**At the bottom of page 8, replace “resulting results” with just “results”.**

**I replaced.**

**I appreciate the thoroughness with which you have constructed the data set. Still, I harken back to my days within a data science classroom where my professor stated that the rule of thumb for whether or not to provide imputations within a model was 10%...in other words, if a variable had more than 10% of its data missing, then you should just drop it from a model altogether. Here, the stakes seem even higher, in that you are building theoretical database for even more researchers to use in the future, based on your imputation strategy. Clearly, from Table 1, you can see that quite a few variables have far more than 10% of the data missing. I do appreciate your efforts in choosing the right imputation strategy here, and I do not take issue with the steps you have taken. However, I would be much more relieved if you could point to similar instances in which researchers have done a complex imputation strategy such as yours, and produced a viable data set that has been replicable and well-utilized by the research community in the past. Since Rubin (1987) is the seminal work on this issue, and you cite it liberally throughout the manuscript, I am tempted to agree with your work here but having precedent would certainly be nice to see.**

**I appreciate your kind comments. The rule of thumb (10% missingness) may well be applicable in the case of single imputation (cold or hot deck imputation). Multiple imputation by design generates multiple copies (I generated 50 copies because some variables had high missing percentage, as suggested by the literature) and is thus better able to incorporate uncertainty underlying missing data. In fact, when missingness is high, using MI is recommended (see these articles: Lee and Huber (2021)** [**https://www.ncbi.nlm.nih.gov/pmc/articles/PMC8426774/**](https://www.ncbi.nlm.nih.gov/pmc/articles/PMC8426774/) **; Janssen et al.2010** [**https://pubmed.ncbi.nlm.nih.gov/20338724/**](https://pubmed.ncbi.nlm.nih.gov/20338724/)**; Pedersen et al. 2017** [**https://www.ncbi.nlm.nih.gov/pmc/articles/PMC5358992/**](https://www.ncbi.nlm.nih.gov/pmc/articles/PMC5358992/)**). According to these and other papers, MI produces less biased estimates among all the corrective procedures. What is important is the number of imputations needed, the justification of the assumptions behind imputation technique, and acceptance/rejection criteria for a variable that is a potential candidate for imputation. I should acknowledge no matter what imputation one may follow; it cannot be 100 percent correct. However, if the process is rigorously followed and an appropriate imputation model is correctly implemented, we can get leased biased results.**

**The precedent dataset (CANA) that I have modeled after my dataset also has missingness on variables ranging from 2% to over 90% (**[**https://www.tandfonline.com/doi/abs/10.1080/2157930X.2011.605871**](https://www.tandfonline.com/doi/abs/10.1080/2157930X.2011.605871)**). This dataset is well-utilized and the paper about this dataset is cited over 130 times.**

**Also see these papers:**

**Janssen et al., 2010** [**https://pubmed.ncbi.nlm.nih.gov/20338724/**](https://pubmed.ncbi.nlm.nih.gov/20338724/)

**Sterne 2009** [**https://www.bmj.com/content/338/bmj.b2393**](https://www.bmj.com/content/338/bmj.b2393) **cited over 4000 times**

**Most of MI datasets are used in medical science because they are least costly, and you do not want to ignore important variation (in subjects) because of missing data.**

On page 19, you make the following statement: “I have conducted a detailed analysis in another article (Khan 2021).” Just a small issue in that appears to be a working paper. It is nitpicky, but it has subtle differences.

**Thank you for pointing this out. The paper (Khan 2021) is now published which I cited here.**

**I genuinely appreciate your service. Sincerely, Author.**

**Reviewer 2: (Key: Green text- Reviewer’s comment; Black text-Author’s response)**

**Summary: This paper proposes a new complete panel dataset with no missing values for the low- and middle-income countries eligible for World Bank’s International Development Association’s support, by using Predictive Mean Matching developed by Rubin (1987). I think that this paper addresses an important issue (data limitation) and contributes to the literature by providing new data set. I think that the author needs to support the pivotal assumptions of the method he or she used.**

**Thank you for your very useful feedback. I have addressed your concerns about assumptions as follows.**

1. **The Multiple Imputation Method requires MAR (Missing at Random) assumption in page 10, according to the author. However, the author didn’t justify this assumption enough in the draft. The author just says “I argue that this rich corpus of data can be employed to explain and predict the missingness pattern for data on other variables, thus justifying the MAR assumption” and “The inclusion of complete identifiers and other auxiliary variables increases the precision of the imputation results for variables exhibiting high missingness and makes the MAR assumption more plausible”. The author also says “Missing At Random (MAR)- Data exhibits MAR if the missingness is due to observed but not unobserved data. In other words, the observed data explains the missingness.”. I don’t understand how merely having rich data set can support the argument that no unobserved data causes the missingness.**

**Thank you for pointing this out. In response to your valuable comments and to address how the data most likely satisfy MAR assumption, I added more text, rewrote several paragraphs, added footnotes 2 and 3, and cited more studies (Rhodes 2012; Fairclough et al., 1998; Castellacci and Natera 2011) in Section 5 under *Step 2- Choice of Specification*. I produce the text (alongside footnotes) for your convenience below:**

***Step2- Choice of Specification: To multiply impute, the choice of a correct multiple imputation specification is necessary. In STATA, either one can employ multivariate normal (MVN) MI or MI by chained equations (MICE).^[1]^ Both these strategies assume a MAR pattern in data before execution.^[2]^ There is no direct way of testing this (and other missingness MCAR and MNAR) assumption(s) illustrated earlier [67]. All one can do is offer a plausible explanation of how the missingness pattern may conform to MAR assumption or how it may not be MNAR and MCAR. Alternatively, one may investigate indirectly, including sensitivity analyses and multiple reliability checks of the imputed dataset, that are far from perfection [67].^[3]^ I argue missingness in LMICs exhibit a MAR pattern. In other words, the missingness pattern in data in LMICs is “somewhat” random (as opposed to “completely random” required by MCAR or “truly systematic” as can be seen in the MNAR pattern). The MAR pattern, by definition, implies that the observed data (in LMICs) can explain and predict missingness [59]. Therefore, to say that a dataset for LMICs has a MAR pattern, a researcher needs access to some available data in these countries.***

***To reiterate, I argue that LMICs have most likely MAR data pattern that is somewhat random but not entirely systematic.*** ***If the missing would have been all systematic (MNAR pattern), then we would not have rich data in such countries on other important economic, geographic, seasonal, and demographic data. Since we have plenty of data for the parameters mentioned earlier in LMICs, the data are not MNAR. On the other hand, if missingness had been completely random (MCAR), i.e., neither missing data nor available data impact missingness in LMICs, the missing pattern in variables would have been uncorrelated. As the missingness pattern in LMICs is correlated in many or some variables, the data are not MCAR. Thus, the data in LMICs are MAR. LMICs can have missing data for various reasons, ranging from poor data infrastructures and meager resources to frequent natural disasters and severe civil conflicts. However, despite missingness in many variables of significance, the availability of rich information on poverty indicators, economic development, literacy rates, and demographics in LMICs can be useful. The propensity of missing values for essential variables (such as science and tech articles, budget allocation to education, and service sector employment, among other variables in the dataset) are systematically linked with the LMICs’ observed data (not the missing data) on GDP, per capita income, and literacy rates variables. For instance, a country’s per capita income and literacy rate relate to a country’s allocation to education expenditure. Thus, I argue that the rich corpus of observed data can be employed to explain and predict the missingness pattern for data on other variables, as required by the MAR assumption.***

a. **It is understandable that it is impossible to test MAR assumption directly. However, the author may be able to find other justification or indirect test to check this assumption.**

**Dear Reviewer: I have offered justification and explanation for the MAR assumption (please see previous response).**

**As mentioned in previous response, MAR assumption is untestable (Rhoads 2012- https://cepa.uconn.edu/wp-content/uploads/sites/399/2015/12/stat-politics-policy2.pdf).** **There maybe some indirect ways but they are also far from perfect (Rhoads 2012).** **One way would be** **to do some other imputation model that requires MNAR assumption and then compare the estimates (and standard errors) from both analyses. Fairclough et al (1998 - https://pubmed.ncbi.nlm.nih.gov/9549823/) conducts sensitivity analyses to obtain comparable results under both MAR and MNAR assumptions and then use this as a basis for the claim that, “The assumption that the data were missing at random (MAR) was adequate to obtain unbiased estimates.” However, it is hard to support this claim.** **Same estimates from different models may not mean unbiased estimates.** **Instead of doing imperfect sensitivity analysis of the dataset, one may compare the analysis results obtained through imputed and observed (incomplete) datasets. The comparison may offer (ex-post) interesting insights about the quality of the dataset.**

**Another way perhaps would be to do reliability checks of the imputed dataset just like the author has performed in this paper (Castallacci and Natera 2011 also did this). If the imputed dataset is reliable, it may imply that MAR assumption was reasonably met and the bias in estimates is most likely mitigated.**

**In this paper, I am not doing the former option for the sake of brevity. I will leave this option for future research (I indicated this in the last paragraph of Conclusion Section). Here I will extend possible explanation alongside reliability checks.**

2. **In page 17, the author says “Furthermore, since all the variables are continuous, differently distributed, and missingness among them is arbitrary, Rubin’s (1987) multiple imputation by chained equations (MICE) best serves this study.”. It may be true that all variables are continuous, differently distributed. However, I don’t see the reason why missingness among them is “arbitrary”.**
   a. **The author may define the meaning of “arbitrary” in this case and why missingness is “arbitrary” in this case.**

**Arbitrary means “not systematic.” This is because of MAR assumption. I have added “somewhat” before “arbitrary” to indicate that the missingness is not truly random as required by MCAR.**

b. **If “arbitrary” means MAR assumption, then the aforementioned sentence (all the variables are continuous, differently distributed, and missingness among them is arbitrary, Rubin’s (1987)….) cannot be meaningful since the author didn’t justify the MAR assumption enough.**

**Please read my previous responses. Now I have clarified the MAR assumption. The MAR assumption is justified because the missingness is not systemic. Had the missing been systemic, we would not have seen other rich data. Missingness is perhaps because of chance events, disasters, civil conflicts, and poor data resources in LMICs. The author carried out imputation (in a fully conditional model) of each and every missing variable (treated as outcome variable) in a series of univariate regression models while treating observed data as predictors in the model.**

3. **In page 23, it says “a health convergence” and “A healthy convergence means that variance between and within iterations is the same”. The author needs a citation.**

**Thank you for pointing this out. I have included citations and further elaborated this part. Part of the paragraph now reads as follows:**

***Therefore, to establish the reliability of the imputation process, I checked for convergence among iterations for imputed variables. Convergence can be checked in a few ways. One way is to plot the mean and variance of the imputed values of different missing variables against the iteration number [Zahid et al., 2021]. For healthy convergence, these plots for m imputed datasets should freely intermingle and there should not be any definite trends [Please see these: Zahid et al., 2021 and*** [***https://stefvanbuuren.name/fimd/sec-algoptions.html***](https://stefvanbuuren.name/fimd/sec-algoptions.html) ***Flexible Imputation of Missing Data by Stef van Buuren, second edition, published by CRC Press, Taylor & Francis Group]. Another way is to examine between and within sequence variance [Ibid]. On healthy convergence, the variance between sequences is no larger than the variance within each individual sequence [Please see these: Zahid et al., 2021;*** [***https://stefvanbuuren.name/fimd/sec-algoptions.html***](https://stefvanbuuren.name/fimd/sec-algoptions.html) ***Flexible Imputation of Missing Data by Stef van Buuren, second edition, published by CRC Press, Taylor & Francis Group; and*** [***https://bookdown.org/mwheymans/bookmi/multiple-imputation.html***](https://bookdown.org/mwheymans/bookmi/multiple-imputation.html)

***By Heymans and Eekhout 2019]. Since the plots for imputed datasets freely intermingled with no definite trend, the convergence pattern of the iterations through which the dataset was generated showed a healthy convergence (S9 Fig.).***

**Thank you so much for your feedback.**

**Editor’s Comment: (Key: Green text- Reviewer’s comment; Black text-Author’s response)**

1. **Please ensure that your manuscript meets PLOS ONE's style requirements, including those for file naming. The PLOS ONE style templates can be found at**

**https://journals.plos.org/plosone/s/file?id=wjVg/PLOSOne_formatting_sample_main_body.pdf and**

[**https://journals.plos.org/plosone/s/file?id=ba62/PLOSOne_formatting_sample_title_authors_affiliations.pdf**](https://secure-web.cisco.com/1UTfjIBUVRSjQxydCCcFNn5GZjtLSYCL4Wftl_fzSURLEXT_5va6G6BgaLliPOspGYBr7KDQcpbfE-m2TtXeniSlTAKmO13qI0mIAHuJ5vYXfWROWUbrQwIzIiwXssH9fMQdhD6I4-8zlEWWlaZhSNSFvbh91TMlkLQqiTWYkyUElKnfjrvGl65BMwMPf_KEmMjU1ovyOegRJ5cb-_Y1MYuRbZJ2_J2avohy53tvFcug1O_8NII6_VeI8HMnBJQBt9DNbmwrpqUZR6AeqW-9TB72YYSL2bPjQfRruVVh8f1FOeVRX_aGUpkkJQAhHw6qGNvLf6NMSMndrNJkcmbZVEaHVKNx1mlN161SGYuytM8B_7jr5smDZ98WyMevc0oUnDT7W1TrBGhsi_v33YobaakV37jjeq00fvLjBEHB-zm0_2nG8Cda3y9iAYPPadxCl/https%3A%2F%2Fjournals.plos.org%2Fplosone%2Fs%2Ffile%3Fid%3Dba62%2FPLOSOne_formatting_sample_title_authors_affiliations.pdf)**.**

**I have ensured to meet the journal’s requirements.**

2**. We noted in your submission details that a portion of your manuscript may have been presented or published elsewhere. [DETAILS AS NEEDED] Please clarify whether this [conference proceeding or publication] was peer-reviewed and formally published. If this work was previously peer-reviewed and published, in the cover letter please provide the reason that this work does not constitute dual publication and should be included in the current manuscript.**

**Figure 2 in this paper is published in another paper that the author wrote (**[**https://www.sciencedirect.com/science/article/pii/S0954349X22000509?casa_token=wSO7CJQrSOUAAAAA:HW9-eLKk80kjAG9oatFxJZOgSSwEgT-lCIQ637Dd4I4AfmUFiN9vnCjlqenUVS5l8s5NLjGYbg**](https://www.sciencedirect.com/science/article/pii/S0954349X22000509?casa_token=wSO7CJQrSOUAAAAA:HW9-eLKk80kjAG9oatFxJZOgSSwEgT-lCIQ637Dd4I4AfmUFiN9vnCjlqenUVS5l8s5NLjGYbg)**)**

**I have included the figure source in the paper now. The author designed the figure himself thus there is no need to seek permission from anyone.**

**Additionally, to improve the paper, I presented it in 2 peer-reviewed conferences. I have included the information in the acknowledgement section. Secondly, a preprint of this paper is up on arXIV in accordance with the PLOS ONE journal standards. Neither of these (preprint, conference presentation, or the figure included with proper reference) constitute formal/dual publication.**

3**. Please note that in order to use the direct billing option the corresponding author must be affiliated with the chosen institute. Please either amend your manuscript to change the affiliation or corresponding author, or email us at plosone@plos.org with a request to remove this option.**

**No change of affiliation.**

**Thank you so much for your service.**

1. One can employ Amelia II in R statistical tool [54]. However, Amelia II assumes normality, which is not the case here.
2. As illustrated in Section 3, missing data patterns or mechanisms are of three kinds: MCAR, MAR, and MNAR. MCAR is the most restrictive assumption (requiring the missingness to be truly random) whereas MNAR is the most relaxed (requiring the missingness to be systematic). MAR is a moderately restricted assumption between these two assumptions that involves some degree of randomness.
3. Apart from the plausible explanation, I offer here, one indirect way to check for MAR assumption is to implement some other imputation model that requires MNAR assumption and then compare the estimates (and standard errors) from both MAR and MNAR analyses (as conducted by [68]). The authors obtained approximately similar estimates in both analyses, thus claiming that their MAR assumption was adequate to get unbiased estimates [68]. However, it is hard to support this claim fully—the same estimates from different models may not mean unbiased estimates.

   In addition to performing an imperfect sensitivity analysis of the dataset, another way would be to do reliability checks of the imputed dataset, just like the author has performed in this paper (the authors of the CANA dataset [31] also performed reliability checks). If the imputed dataset is reliable, it may imply that the MAR assumption was reasonably met and the bias in estimates is most likely mitigated.

   Another indirect way (ex-post) would be to compare the analysis results obtained through imputed (under MAR) and observed (incomplete) datasets, which may offer interesting retrospective insights about the quality of the dataset.
