# Supplementary material for: Estimating a panel MSK dataset for comparative analyses of national absorptive capacity systems, economic growth, and development in low and middle income countries
Source: PLoS One. 2022 Oct 20;17(10):e0274402. doi: 10.1371/journal.pone.0274402 (PMC9584427; doi:10.1371/journal.pone.0274402)
Supplement: Attachment — Submitted filename: Referee report.docx [file pone.0274402.s010.docx]

Referee report: Estimating a new panel MSK dataset for comparative analyses of national absorptive capacity systems, economic growth, and development in low and middle income economies

Summary: This paper proposes a new complete panel dataset with no missing values for the low- and middle-income countries eligible for World Bank’s International Development Association’s support, by using Predictive Mean Matching developed by Rubin (1987). I think that this paper addresses an important issue (data limitation) and contributes to the literature by providing new data set. I think that the author needs to support the pivotal assumptions of the method he or she used.

1. The Multiple Imputation Method requires MAR (Missing at Random) assumption in page 10, according to the author. However, the author didn’t justify this assumption enough in the draft. The author just says “I argue that this rich corpus of data can be employed to explain and predict the missingness pattern for data on other variables, thus justifying the MAR assumption” and “The inclusion of complete identifiers and other auxiliary variables increases the precision of the imputation results for variables exhibiting high missingness and makes the MAR assumption more plausible”. The author also says “Missing At Random (MAR)- Data exhibits MAR if the missingness is due to observed but not unobserved data. In other words, the observed data explains the missingness.”. I don’t understand how merely having rich data set can support the argument that no unobserved data causes the missingness.
   a. It is understandable that it is impossible to test MAR assumption directly. However, the author may be able to find other justification or indirect test to check this assumption.
2. In page 17, the author says “Furthermore, since all the variables are continuous, differently distributed, and missingness among them is arbitrary, Rubin’s (1987) multiple imputation by chained equations (MICE) best serves this study.”. It may be true that all variables are continuous, differently distributed. However, I don’t see the reason why missingness among them is “arbitrary”.
   a. The author may define the meaning of “arbitrary” in this case and why missingness is “arbitrary” in this case.
   b. If “arbitrary” means MAR assumption, then the aforementioned sentence (all the variables are continuous, differently distributed, and missingness among them is arbitrary, Rubin’s (1987)….) cannot be meaningful since the author didn’t justify the MAR assumption enough.
3. In page 23, it says “a health convergence” and “A healthy convergence means that variance between and within iterations is the same”. The author needs a citation.
